# Leveraging hand-crafted radiomics on multicenter FLAIR MRI for predicting disability worsening in people with multiple sclerosis

**DOI:** 10.3389/fnins.2025.1610401

**Published:** 2025-10-29

**Authors:** Hamza Khan, Henry C. Woodruff, Diana L. Giraldo, Lorin Werthen-Brabants, Shruti Atul Mali, Sina Amirrajab, Edward De Brouwer, Veronica Popescu, Bart Van Wijmeersch, Oliver Gerlach, Jan Sijbers, Liesbet M. Peeters, Philippe Lambin

**Affiliations:** ^1^University MS Center, Biomedical Research Institute (BIOMED), Hasselt University, Diepenbeek, Belgium; ^2^Data Science Institute (DSI), Hasselt University, Diepenbeek, Belgium; ^3^The D-Lab, Department of Precision Medicine, GROW – Research Institute for Oncology and Reproduction, Maastricht University, Maastricht, Netherlands; ^4^Department of Radiology and Nuclear Imaging, GROW – Research Institute for Oncology and Developmental Biology, Maastricht University Medical Centre, Maastricht, Netherlands; ^5^imec-Vision Lab, University of Antwerp, Antwerp, Belgium; ^6^μNEURO Research Centre of Excellence, University of Antwerp, Antwerp, Belgium; ^7^SUMO Group, IDLab, Ghent University – imec, Ghent, Belgium; ^8^ESAT-STADIUS, KU Leuven, Leuven, Belgium; ^9^Noorderhart, Rehabilitation and MS Center, Pelt, Belgium; ^10^Academic MS Center Zuyd, Department of Neurology, Zuyderland Medical Center, Sittard-Geleen, Netherlands; ^11^School for Mental Health and Neuroscience, Maastricht University, Maastricht, Netherlands

**Keywords:** multiple sclerosis, radiomics, magnetic resonance imaging, FLAIR MRI, white matter lesions, disability worsening, machine learning

## Abstract

**Background:**

Multiple sclerosis (MS) is an autoimmune disease of the central nervous system, leading to varying degrees of functional impairment. Conventional tools, such as the Expanded Disability Status Scale (EDSS), lack sensitivity to subtle disease worsening. Radiomics provides a quantitative imaging approach to address this limitation. This study applied machine learning (ML) and radiomics features from T2-weighted Fluid-Attenuated Inversion Recovery (FLAIR) magnetic resonance imaging (MRI) to predict disability worsening in MS.

**Methods:**

A retrospective analysis was performed on real-world data from 247 PwMS across two centers. Disability worsening was defined as a change in EDSS over two years. FLAIR MRIs underwent preprocessing and super-resolution reconstruction to enhance low-resolution images. White matter lesions (WML) were segmented using the Lesion Segmentation Toolbox (LST), and tissue segmentation was performed using sequence Adaptive Multimodal Segmentation. Radiomics features from WML and normal-appearing white matter (NAWM) were extracted using Pyradiomics, harmonized with Longitudinal ComBat, followed by recursive feature elimination for feature selection. Elastic Net, Balanced Random Forest (BRFC), and Light Gradient-Boosting Machine (LGBM) models were trained and evaluated.

**Results:**

The LGBM model with harmonized radiomics and clinical features outperformed the clinical-only model, achieving a test area under the precision-recall curve (PR AUC) of 0.20 and a receiver operating characteristic area under the curve (ROC AUC) of 0.64. Key predictive features, among others, included Gray-Level Co-Occurrence Matrix (GLCM) maximum probability (WML) and Gray-Level Dependence Matrix (GLDM) dependence non-uniformity (NAWM). However, short-term longitudinal changes showed limited predictive power (PR AUC = 0.11, ROC AUC = 0.69).

**Conclusion:**

These findings highlight the potential of ML-driven radiomics in predicting disability worsening, warranting validation in larger, balanced datasets and exploration of advanced deep learning approaches.

## Highlights

- Machine learning improves the prediction of disability worsening in multiple sclerosis.
- Radiomics can capture subtle, diffuse changes in MS worsening from FLAIR MRI.
- Super-resolution reconstruction enhances radiomics analysis of low-resolution MRIs.

## Introduction

**Figure 1:**
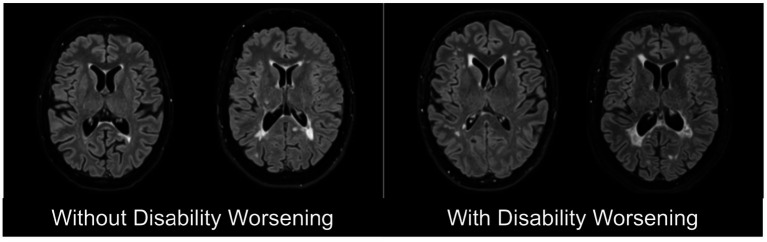
Representative T2-weighted Fluid-attenuated Inversion Recovery (FLAIR) magnetic resonance imaging (MRI) scans of people with multiple sclerosis (PwMS) without disability worsening (left) and with disability worsening (right).

Multiple Sclerosis (MS) is a chronic neuroinflammatory autoimmune disease of the central nervous system (CNS) characterized by demyelination and axonal damage, resulting in varying degrees of disability worsening (Calabresi, 2004). Despite advances in disease-modifying therapies, predicting disability remains difficult due to the heterogeneity of disease courses and incomplete understanding of their pathophysiology (Thompson et al., 2018; Tilling et al., 2016). Accurate prediction has critical clinical implications, as it can optimize monitoring schedules, inform therapeutic decisions, and enable more effective patient stratification for clinical trials, thereby improving outcomes, reducing healthcare burden, and accelerating therapy development (Dennison et al., 2018; Inojosa et al., 2021). Figure 1 illustrates magnetic resonance imaging (MRI) scans of PwMS with and without disability worsening.

The primary objective of this study was to predict disability worsening in PwMS over a two-year period. Disability worsening, derived from changes in the Expanded Disability Status Scale (EDSS) (Kurtzke, 1983), corresponds to the worsening of physical capabilities. In clinical practice, EDSS and MRI scans are commonly used to diagnose and assess the course of MS disease (Wattjes et al., 2021). However, both have their respective limitations. EDSS is prone to inter-rater variability and lacks sensitivity to short-term changes, while MRI features, such as the number and volume of white matter lesions (WML) or their gadolinium enhancement, explain only part of clinical outcomes (Uitdehaag, 2018). Moreover, these measures fail to track the diffuse pathological changes in the gray matter (GM) and normal-appearing white matter (NAWM) (Wattjes et al., 2021; Davda et al., 2019; Treaba et al., 2019). Therefore, there is an unmet clinical need for more sensitive and specific biomarkers to predict disability worsening in PwMS.

An image quantification approach, such as radiomics, can potentially overcome this gap (Pontillo et al., 2021). By extracting high-dimensional quantitative features related to shape, intensity, and texture from regions of interest (ROIs), radiomics can characterize subtle changes and correlate them with clinical endpoints (Gillies et al., 2016; Lambin et al., 2012; Lambin et al., 2017; Rogers et al., 2020). Radiomics has shown promising results in different disease domains, including oncology (Lambin et al., 2017; van Timmeren et al., 2017), Alzheimer’s disease (Feng et al., 2018; Li et al., 2019), and epilepsy (Liu et al., 2018). Similarly, in MS, radiomics features can potentially become clinically relevant noninvasive disease biomarkers for MS disease worsening (Lavrova et al., 2021). Some of these features include cortical lesion volume (Calabrese et al., 2010), spinal and brain volume atrophy (Kearney et al., 2015; Storelli et al., 2018), microstructural damage of NAWM (Moll et al., 2011), and the structural changes in the GM (Pontillo et al., 2019).

Given the limitations in EDSS and MRI, training advanced machine learning (ML) techniques with radiomics features holds promise for developing predictive models. By analyzing high-dimensional imaging data, ML models can potentially capture and quantify imaging biomarkers associated with MS worsening, offering a more robust, objective, and sensitive prediction of disability.

In this study, we used ML to predict two-year EDSS-based disability worsening in PwMS using real-world data (RWD). Catering to the unmet clinical need to identify a noninvasive quantitative biomarker that is sensitive to disability worsening in MS, we hypothesize the following:

- Radiomics-based ML models can outperform models relying solely on clinical variables to predict disability worsening in PwMS.
- Radiomics features from MRI can predict disability worsening (2 years) in PwMS.
- Short-term changes (6 months) in radiomics features can predict disability worsening in PwMS.

## Materials and methods

### Inclusion criteria

To ensure the longitudinal tracking of disability worsening, inclusion criteria were designed to capture both clinical and imaging data at consistent intervals. This approach was necessary to align MRI scans with corresponding EDSS measurements over time, providing a comprehensive assessment of disease worsening. Subjects with a confirmed diagnosis of MS, at least a two-year longitudinal EDSS score trajectory, and at least one baseline and one follow-up MRI scan were included in the study. To achieve this, anchor dates were defined as fixed reference points based on visits (e.g., MRI acquisition dates) and fixed time points (e.g., 6 months and 2 years after the initial visit). Temporal windows were specified around the anchor dates to select the follow-up MRI. The three temporal windows were defined as:

- T0 (initial visit): The anchor date for T0 is the MRI acquisition date. The closest EDSS measurement within a 6-month window before or a 3-month window after the T0 anchor date was selected as the baseline score EDSS_T0.
- T1 (short-term follow-up): The anchor date for T1 was set exactly 6 months after the T0 anchor date. The T1 window spanned 3 months before and 3 months after the T1 anchor date. Thus, the MRI session at T1 was selected to calculate short-term changes.
- T2 (long-term follow-up): The anchor date for T2 was set 2 years after the T0 anchor date. The T2 window spanned from 3 months before to 1 year after the T2 anchor date. The closest EDSS measurement to the T2 anchor date was selected as EDSS_T2. No MRI was included in this window, as T2 was based solely on EDSS to assess two-year disability worsening.

**Figure 2:**
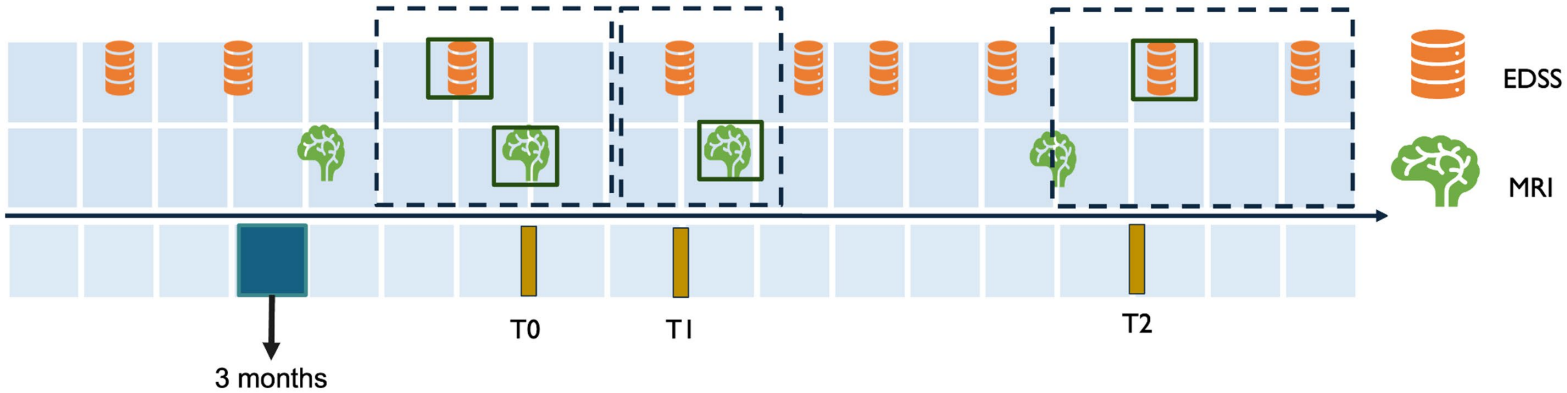
Temporal alignment of EDSS and MRI data for inclusion criteria. This figure illustrates the temporal windows used to align MRI and EDSS data for assessing disability worsening. T0 represents the baseline MRI session, with the closest EDSS measurement selected within a window of 3 months after or 6 months before the MRI. T1 corresponds to the short-term follow-up 6 months after T0, with a 3-month window before and after the T1 anchor date for EDSS and MRI selection. T2 represents the two-year follow-up 2 years after T0, with the EDSS measurement selected within a window of 3 months before 1 year and after the T2 anchor date.

This structure enabled the consistent alignment of MRI data and EDSS scores at the initial visit (T0) and short-term follow-up (T1), while two-year worsening (T2) was assessed using EDSS alone. Multiple MRI sessions and their corresponding EDSS scores were included for some subjects, with a range of 2 to 8 MRIs per subject. Each MRI session contributed to the analysis and was treated as a separate observation. Furthermore, MRI sessions from the same subject were grouped during partitioning into training, validation, and test sets to avoid potential data leakage. An overview of the temporal windows is illustrated in Figure 2.

### Endpoint definition

The primary endpoint of this study was two-year disability worsening, and its definition was adapted from previous work (Kalincik et al., 2017). Disability worsening was determined by comparing EDSS scores between T0 and T2. A subject was considered worsened if the change in EDSS met the following criteria:

- EDSS_T2 – EDSS_T0 ≥ 1.5 for patients with EDSS_T0 = 0.
- EDSS_T2 – EDSS_T0 ≥ 1.0 for patients with EDSS_T0 ≤ 5.5.
- EDSS_T2 – EDSS_T0 ≥ 0.5 for patients with EDSS_T0 > 5.5.

### Modeling pipeline

The pipeline consists of three main stages: image processing, feature processing, and modeling. The image processing stage preprocessed MRI data through reorientation, denoising, bias field correction, super-resolution reconstruction, and segmentation of WML and NAWM. The feature processing stage focused on extracting radiomics features, harmonizing them to reduce inter-scanner variability, and removing redundant features. Finally, the modeling stage explored four predictive approaches—clinical, baseline imaging, longitudinal imaging, and combined—by employing feature selection, ML models, and validation techniques to evaluate predictive performance. As described later in the section “Data Partitioning,” the datasets were shuffled and split into training, validation, and testing datasets. Feature harmonization, reduction, selection, model hyperparameter optimization, and model selection were performed exclusively on the training and validation datasets to avoid data leakage.

### Image processing

#### MRI data

Our study used pseudonymized longitudinal MRI data collected retrospectively from two medical centers: the Rehabilitation and MS Center of Noorderhart in Pelt, Belgium (DS1) and Zuyderland Medical Center in Sittard, Netherlands (DS2). The study has been approved by the ethical commission of the University of Hasselt (CME2019/046) and the Medical Ethics Review Committee of Zuyderland and Zuyd University of Applied Sciences (METCZ20200167). No consent to participate was required, given the pseudonymized and retrospective nature of the study. Both DS1 and DS2 have been used for the first time in this study. They are private datasets consisting of T2-weighted Fluid-attenuated Inversion Recovery (FLAIR) MRI scans and clinical data, including age, gender, and EDSS scores, collected during routine clinical follow-ups. After applying the inclusion criteria, a total of 149 subjects from DS1 and 98 subjects from DS2 were included in the analysis. The details of the dataset are mentioned in Table 1.

**Table 1:** Datasets summary details.

Dataset	DS1	DS2	*p*-value
Participants (*n*)	149	98	–
Sessions	630	184	–
Age in years at baseline (± SD)	42.9 ± 11.7	37.4 ± 10.2	0.015*
Female to Male ratio	3.2:1	3.3:1	0.983
EDSS at baseline (IQR, 25th–75th percentile)	2.0 (1.5–3.0)	2.5 (1.5–4.0)	0.242
Acquisition date range	2010–2017	2008–2019	–
Worsening (%)	5.5%	13%	0.087

This table summarizes the cohort characteristics of DS1 and DS2. The *p*-values for continuous variables (Age in years at baseline and EDSS at baseline) were calculated using two-sample *t*-tests, while *p*-values for categorical variables (Female-to-Male ratio and Worsening %) were calculated using chi-square tests. A *p*-value <0.05 is marked with an asterisk (*) and indicates a statistically significant difference. Continuous variables are summarized as mean ± standard deviation (SD) (Age) or median Interquartile range (IQR) (EDSS) as appropriate.

The acquisition protocol for T2-weighted FLAIR varied within and across the two datasets. Images in DS1 were acquired using the same scanner (Philips Achieva 1.5 T) with three different protocols depending on the date. Between 2010 and 2015, the MRI session included two orthogonal multi-slice T2-W FLAIRs acquired with axial and sagittal slice orientations and a slice spacing of 6 mm (Protocol A). Between 2015 and 2017, three orthogonal images were acquired with a slice spacing of 3 mm (Protocol B). In 2017, acquisition sessions included a fast high-resolution 3D T2-W FLAIR with a voxel size of 0.98 mm x 0.98 mm x 0.6 mm (Protocol C), and most of them also included a structural T1-W MRI (Protocol CsT1). Images in DS2 were acquired using nine different scanners with varying protocols, including low-resolution multi-slice and high-resolution 3D acquisitions, with spacing between slices ranging from 0.8 mm to 7 mm. Sessions in DS2 were categorized similarly to sessions in DS1, based on the number and resolution of acquired T2-W FLAIR images. Sessions with a 3D high-resolution image were classified as Protocol C, while those with two orthogonal multi-slice low-resolution images were classified as Protocol A. No sessions contained three orthogonal images, and 29 sessions with only one low-resolution image were classified as Protocol D. Detailed information about T2-W FLAIR MRI acquisition protocols for both datasets is provided in Appendix A.

#### MRI pre-processing

The preprocessing of MRI data aimed at harmonizing and enhancing the image quality before radiomics feature extraction. First, all MRI images were denoised using adaptive non-local means (Manjón et al., 2010), and N4 bias-field correction (Tustison et al., 2010) was applied to mitigate low-frequency intensity inhomogeneities in MRI images caused by magnetic field distortions. This correction aims to improve the accuracy of segmentation and feature extraction, as it reduces the impact of scanner heterogeneity (Tustison et al., 2010).

For protocols with low-resolution images (A, B, and D), we applied “perceptual super-resolution in multiple sclerosis” (PRETTIER) (Giraldo et al., 2024), a super-resolution approach designed to enhance the through-plane resolution of multi-slice structural MRIs containing MS lesions. Since protocols A and B have multiple low-resolution FLAIR images per session, we applied PRETTIER to each image and aligned and combined the outputs following an iterative approach. This technique improves spatial resolution, which is important for downstream radiomics analysis and segmentation tasks. Reconstruction was not performed on protocol C since it had high-resolution FLAIR.

Next, we applied the Sequence Adaptive Multimodal Segmentation (SAMSEG) method for whole-brain segmentation on all FLAIR protocols across DS1 and DS2 (Cerri et al., 2021). SAMSEG is a previously validated segmentation tool designed to segment 41 anatomical brain structures (see Appendix B) from MRI and is fully adaptive to different MRI contrasts and scanners, making it particularly suitable for multi-center datasets like ours. SAMSEG was used to segment, among others, the normal-appearing white matter (NAWM), gray matter (GM), thalamus, and cerebrospinal fluid (CSF).

**Figure 3:**
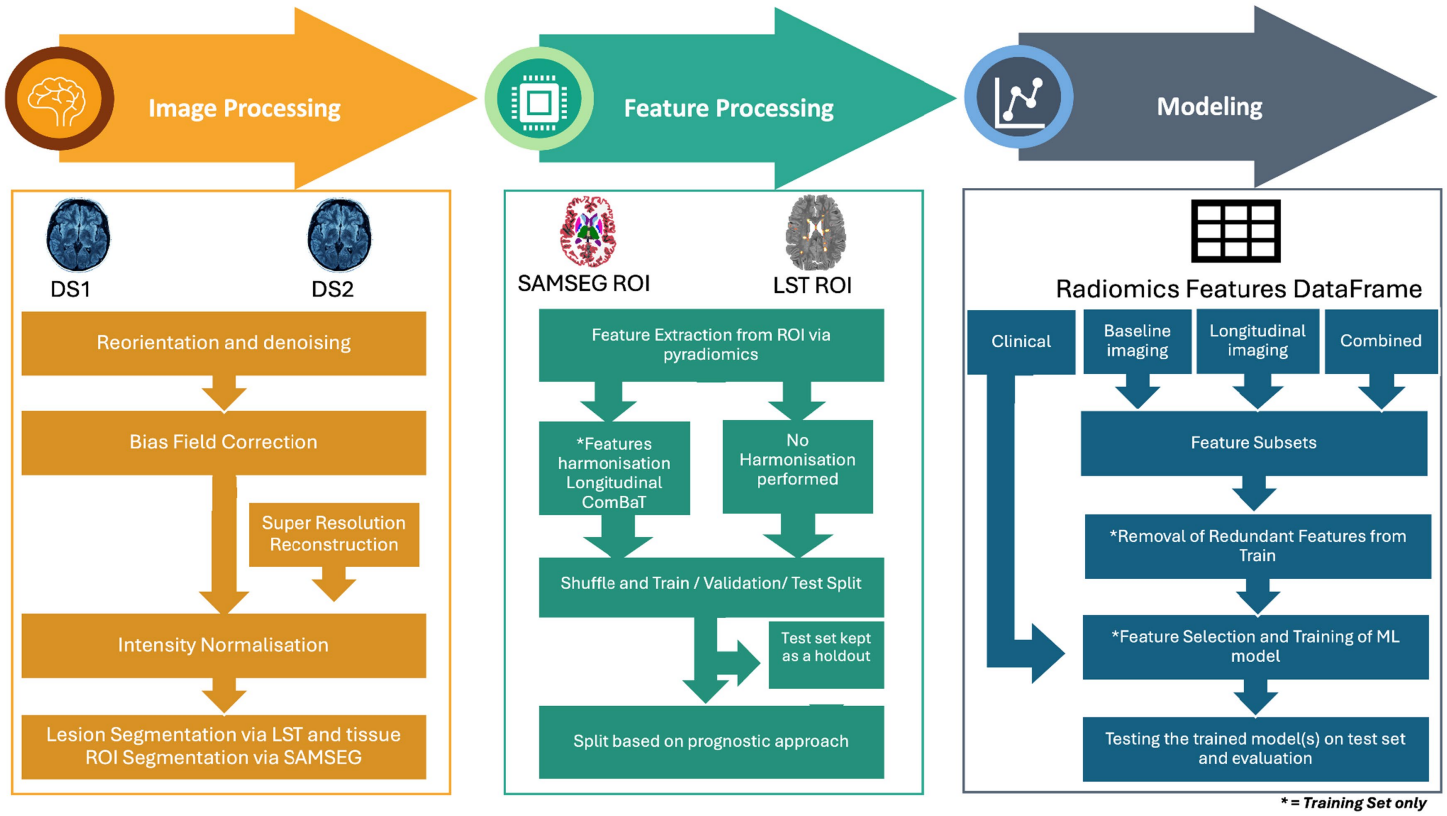
Overview of the methodology for MRI-based disability worsening prediction in multiple sclerosis. The pipeline consists of three main stages: image processing, feature processing, and modeling. Image processing involves image pre-processing steps such as reorientation and denoising, bias field correction, intensity normalization, and lesion/tissue segmentation using LST and SAMSEG. Super-resolution reconstruction was applied to low-resolution images. Feature processing includes the extraction of radiomics features from regions of interest (ROI) and harmonization using longitudinal ComBat to address inter-scanner variability. Features were then divided into harmonized and non-harmonized datasets. The feature sets extracted from DS1 and DS2 were then shuffled and divided into training, validation, and test sets. Modeling stage includes the division of dataset according to four prognostic approaches clinical, baseline imaging, longitudinal imaging and combined. This was followed by further subdividing each approach into feature subsets and subsequent removal of feature reduction, feature selection, and training of a machine learning model, and finally evaluating the best model on the test set.

Lesions were segmented using the lesion prediction algorithm (Schmidt, 2017) as implemented in LST toolbox version 1.2.3^1^ for SPM8.^2^ This algorithm uses a pre-trained logistic regression model to generate lesion probability estimates at each voxel. These lesion probability estimates were thresholded at 0.1 to create white matter lesion (WML) masks. The flowchart of the entire pipeline is shown in Figure 3.

### Feature processing

Besides having masks as the outcome of segmentation, anatomical and lesion volumes were also obtained using SAMSEG and LST. These volumes were subsequently normalized by intracranial volume to ensure comparability across subjects. Per-image adaptive histogram matching (Pizer et al., 1987) was then performed to normalize the intensity distributions of all the skull-stripped FLAIR images, ensuring consistency in intensity values across images.

For this study, high-dimensional radiomics features were extracted from two regions of interest (ROI): the NAWM and the WML. The WML mask was subtracted from the segmented WM mask to generate the NAWM mask. These binary masks, along with the intensity-normalized FLAIR images, were used to compute the corresponding radiomics features for further analysis.

#### Feature extraction

Radiomics features from the ROIs were extracted using Pyradiomics 2.20 (van Griethuysen et al., 2017) with Python 3.7.1. The extracted radiomics features comprised six classes, including shape (Lorensen and Cline, 1987), first-order statistics (FO), gray-level co-occurrence matrix (GLCM) (Haralick et al., 1973), gray-level run length matrix (GLRLM) (Galloway, 1975), gray-level size zone matrix (GLSZM) (Thibault et al., 2013), and gray-level dependence matrix (GLDM) (Sun and Wee, 1983). Gray-level features were calculated by discretizing the images into 50 bins, which aligns with the recommendations by the Image Biomarker Standardization Initiative (IBSI) (Zwanenburg et al., 2018) and in the documentation of Pyradiomics (van Griethuysen et al., 2017). The details of the number of features extracted per class are available in Appendix K.

#### Feature harmonization

Given the diversity of MRI acquisition protocols and the longitudinal heterogeneity of DS1 and DS2, harmonization of the radiomics features between different protocols was performed using longitudinal ComBat (Beer et al., 2020). This technique was applied to improve the comparability of features across the datasets by minimizing inter- and intra-site variability, as well as temporal variations in MRI, which can stem from the acquisition protocol. The principal component analysis (PCA) visualization of the features from DS1 and DS2 before and after harmonization is illustrated in Appendix H. Harmonization coefficients were calculated in the training dataset only and later applied to the testing and validation datasets.

To ensure a comprehensive evaluation, all subsequent steps were conducted separately on both harmonized and non-harmonized datasets.

#### Dataset partitioning

The DS1 and DS2 were shuffled and were then split into training (60%), validation (20%), and test (20%) sets. The splitting was performed using a stratified shuffled split using scikit-learn (Pedregosa et al., 2011). This ensured two things: (1) the distribution of subjects with worsening disability was stratified across the sets as evenly as possible and (2) sessions of the same subject were kept within the same dataset, preventing data leakage.

#### Prognostic approaches

For this study, four distinct prognostic approaches were defined. First, we adopted a “clinical” approach, focusing solely on routinely available clinical variables such as gender, clinical age (in years), and EDSS at T0. The reason for analyzing this approach separately was to evaluate the predictive value of clinical features alone, independent of radiomics features, and it served as a baseline for comparison with radiomics-based approaches.

Since our study also aimed to capture the predictive capability of both baseline features and short-term feature changes in MRI data for disability worsening, we further defined three distinct radiomics-based prognostic approaches. For extracting radiomics features, we used all sessions for each subject with the disability worsening label corresponding to that session, labeling this as the “baseline imaging” approach. The second approach, “longitudinal imaging,” focused on short-term changes in all radiomics features relative to the baseline features. This is calculated by dividing the difference between T1 and T0 and dividing it by T0 (Heidt et al., 2024). In this case, the disability worsening label corresponded to the one assigned at T0. Finally, to assess whether combining baseline imaging and longitudinal imaging could improve predictive power, we integrated features used in longitudinal imaging and baseline imaging approaches, referring to this as the “combined” approach, with the disability worsening label taken at T0.

### Modeling

#### Feature subsets

To evaluate further whether radiomics features alone or with clinical data can predict to disability worsening, we divided the features in baseline imaging, longitudinal imaging, and combined prognostic approaches into the following subsets:

- Radiomics volume features: Regional and lesion volumes derived from SAMSEG and LST (normalized by intracranial volume).
- Radiomics features without volumes: Features extracted exclusively using Pyradiomics (e.g., shape, FO, GLCM, GLRLM, GLSZM, and GLDM).
- Radiomics features: A combination of both the selected radiomics volume features and radiomics features without volumes.
- Radiomics and clinical features: A combination of the selected radiomics features with clinical data, including gender (female), clinical age (in years), and EDSS at T0. This was done to test whether radiomics features combined with clinical features enhance the predictive power of the model.

#### Feature selection

Feature reduction and selection were not applied to the clinical-only dataset; however, for the radiomics-based prognostic approaches, feature reduction was performed separately on the training set for both the harmonized and non-harmonized data analysis pipelines. This includes the four feature subsets, and the aim was to eliminate redundant and non-informative features. Initially, all features were normalized using StandardScaler from scikit-learn (Pedregosa et al., 2011), and then pairwise Spearman correlation was computed for the entire feature set. Features that exhibited a Spearman correlation coefficient greater than 0.9 were considered highly correlated. From each pair of intercorrelated features, the one with the higher average Spearman correlation across all other features was flagged for removal. To ensure stability in the selection process, a bootstrapping approach was used: in each iteration, stratified subsamples were generated based on the outcome, and the intercorrelated features were recalculated. Features that appeared as candidates for removal in 50% or more of the bootstrap iterations were discarded from the final dataset, resulting in a robust set of non-intercorrelated features for further analysis.

Recursive Feature Elimination with 20-fold cross-validation (RFECV) was applied on the non-intercorrelated features across each approach and feature subset. Given the imbalance in the training dataset, with only 6.9% of subjects showing worsening disability, we used a Balanced Random Forest Classifier (BRFC) as an estimator within RFECV to account for this imbalance. Stratified Shuffle Split cross-validation was applied to maintain class proportion across folds, and the precision score was chosen as the evaluation metric to prioritize features that improve the precision of disability worsening prediction.

#### Selection of classification model

We used three ML models, Elastic Net (logistic regression), BRFC, and Light Gradient-Boosting Machine (LGBM), to evaluate each prognostic approach and the feature subsets it entails. In an attempt to make the models robust, we employed Optuna to optimize the hyperparameters of each model. Optuna is a framework for efficient hyperparameter tuning using Bayesian optimization (Akiba et al., 2019). Moreover, a 20-fold cross-validation alongside a stratified shuffle split was implemented during model hyperparameter tuning as well. This approach ensured stability by iteratively training and testing models across different subsets of the training data, helping to enhance the generalizability of the predictions. The model that had the best area under the precision-recall curve (PR AUC) on the validation set, per feature subset, was subsequently tested on the test set to evaluate the generalizability of the feature subset per approach. The PR AUC curve was used to select the best-performing models on the validation set, as it emphasizes the trade-off between precision (positive predictive value) and recall (sensitivity), making it especially suited for imbalanced datasets, like ours, where worsening cases are sparse. In addition to the PR AUC curve, the area under the receiver operating characteristic curve (ROC AUC) was also used to give an insight into the discriminative capabilities of the models. For PR AUC, a value significantly above the prevalence of the positive class indicates meaningful performance. For ROC AUC, a value of 0.5 indicates random performance, and higher values reflect better discrimination (Çorbacıoğlu and Aksel, 2023).

To get an actual picture of the sensitivity and specificity of the models, we used Youden’s index (J) to calculate the optimal threshold for binary classification (Youden, 1950). Furthermore, to validate whether the results generated by our models are not a result of random chance, we also conducted permutation analysis by shuffling the outcome variable, i.e., the disability worsening, and re-running model training. The permutation was performed 50 times, and the models were then subsequently evaluated.

To interpret the predictions of the ML models, SHapely Additive exPlanations (SHAP) (Lundberg et al., 2019) were employed. SHAP values were computed for the features of selected models, followed by the generation of summary plots to visualize feature importance and their impact on predictions. The plots ranked the features by their mean absolute SHAP values and their respective effect on the likelihood of disability worsening.

Moreover, the Radiomics Quality Score (RQS) framework was followed to ensure methodological rigor and adherence to radiomics standards (Lambin et al., 2017). The CLEAR (Checklist for Evaluating the Reporting of AI in Radiology) checklist was also used to evaluate the transparency and reproducibility of the ML pipeline, ensuring clarity and alignment with best practices for AI reporting (Kocak et al., 2023).

## Results

### Cohort characteristics

By combining and shuffling DS1 and DS2, a total of 247 PwMS were included in this study. The 247 participants were divided, using stratified shuffled split, into training (*n* = 148), validation (*n* = 49), and test (*n* = 50) sets. Subjects in the training set had an average age of 41.32 years (±12.13), while those in the validation and test sets had averages of 44.79 years (±10.92) and 38.52 years (±10.25), respectively. The female-to-male ratio remained almost consistent across sets at approximately 3.3:1. Worsening disability was observed in 6.9% of the training set, 9.8% of the validation set, and 6.0% of the test set. The summary of cohort characteristics for shuffled datasets is outlined in Table 2.

**Table 2:** Characteristics of the training, validation, and test datasets.

Dataset	Training set	Validation set	Test set	*p*-value
Participants	148	49	50	–
Sessions	470	166	178	–
Age in years at baseline	41.32 ± 12.13	44.79 ± 10.92	38.52 ± 10.25	Training vs. Validation – 0.04* Validation vs. Test – 0.01* Training vs. Test – 0.37
Female to Male ratio	3.2:1	3.3:1	3.3:1	Training vs. Validation – 0.98 Validation vs. Test – 0.99 Training vs. Test – 0.99
EDSS at baseline (IQR, 25th–75th percentile)	2.0 (1.5–3.0)	2.5 (1.5–4.0)	2.0 (1.5–3.0)	0.008*
Percentage Split (of total)	60%	20%	20%	–
Acquisition date range	2008–2019	2010–2019	2010–2019	–
Worsening (%)	6.9%	9.8%	6.0%	Training vs. Validation – 0.67 Validation vs. Test – 0.58 Training vs. Test – 0.84

This table summarizes the cohort characteristics for the training, validation, and test datasets. The *p*-values indicate comparisons between datasets (Training vs. Validation, Validation vs. Test, and Training vs. Test). For EDSS at baseline, comparisons were performed using the Kruskal–Wallis test with Dunn’s post-hoc tests and Benjamini–Hochberg correction for multiple comparisons. For Age in years at baseline, two-sample *t*-tests were applied. Female-to-Male ratio and Worsening percentage were compared using chi-square tests. A *p*-value <0.05 is marked with an asterisk (*) and indicates a statistically significant difference.

The longitudinal imaging approach resulted in a dataset reduction of unique participants and sessions because it was constructed by calculating feature differences between T0 and T1 sessions. The characteristics of the delta datasets are summarized in Table 3.

**Table 3:** Characteristics of longitudinal imaging approach training, validation, and test datasets.

Dataset	Training set	Validation set	Test set
Participants	97	36	33
Sessions	161	63	70
Worsening (%)	5.5%	6.3%	7.1%

### Feature selection

Tissue segmentation using SAMSEG produced 41 anatomical features (volumes only) from different brain regions. Out of 41, two features, “unknown volumes” and “fifth ventricle volume,” were dropped due to their negligible size in the MRI. The remaining 39 features, along with two lesion-specific features from LST—namely, the number of lesions and lesion volume—constituted the radiomics volume feature subset. In instances where LST failed to provide the lesion volume feature, the SAMSEG-derived WML volume was used as a substitute. Additionally, high-dimensional radiomics features extracted using Pyradiomics yielded a total of 200 features for the radiomics features without the volumes subset.

Subsequently, per the prognostic approach, intercorrelated features were dropped, the details of which are summarized in Appendix C. All the unique retained non-intercorrelated features underwent RFECV to get the optimum number of features for downstream ML analysis. The feature subsets selected by RFECV for the best-performing models per approach are shown in Table 4.

**Table 4:** Selected features from the best-performing models for disability worsening prediction.

Approach	Harmonization	Feature subset	Number of features	Selected features
Clinical	Not applicable	Clinical only	3	EDSS_T0, clinical age in years, gender (female)
Baseline imaging	Harmonized (LongCombat)	Radiomics and clinical features	10	GLRLM run variance (WML), GLCM maximum probability (WML), first order kurtosis (NAWM), left lateral ventricle volume, GLDM dependence non uniformity (NAWM), right amygdala volume, GLSZM large area low gray level emphasis (WML), EDSS_T0, clinical age in years, gender (female)
	Non harmonized	Radiomics and clinical features	13	GLRLM run variance (WML), left thalamus volume, left lateral ventricle volume, first order minimum (WML), right amygdala volume, right accumbent area volume, right thalamus volume, left pallidum volume, GLSZM size zone non uniformity (WML), shape minor axis length (NAWM), EDSS_T0, clinical age in years, gender (female)
Longitudinal imaging	Harmonized (LongCombat)	Radiomics volume features	1	delta right cerebellum cortex volume
	Non harmonized	Radiomics features	3	delta GLCM difference entropy (WML), delta GLDM gray level non uniformity (WML), and delta left choroid plexus volume
Combined	Harmonized (LongCombat)	Radiomics features	6	left thalamus volume, delta left cerebellum cortex volume, delta right hippocampus volume, delta right thalamus volume, right thalamus volume, delta GLSZM large area high gray level emphasis (NAWM)
	Non harmonized	Radiomics and clinical features	11	delta GLSZM gray level non uniformity (NAWM), left thalamus volume, delta GLDM large dependence high gray level emphasis (NAWM), right thalamus volume, brain stem volume, delta right thalamus volume, delta right caudate volume, EDSS_T0, clinical age in years, delta clinical age in years, gender (female)

### Machine learning models performance

**Figure 4:**
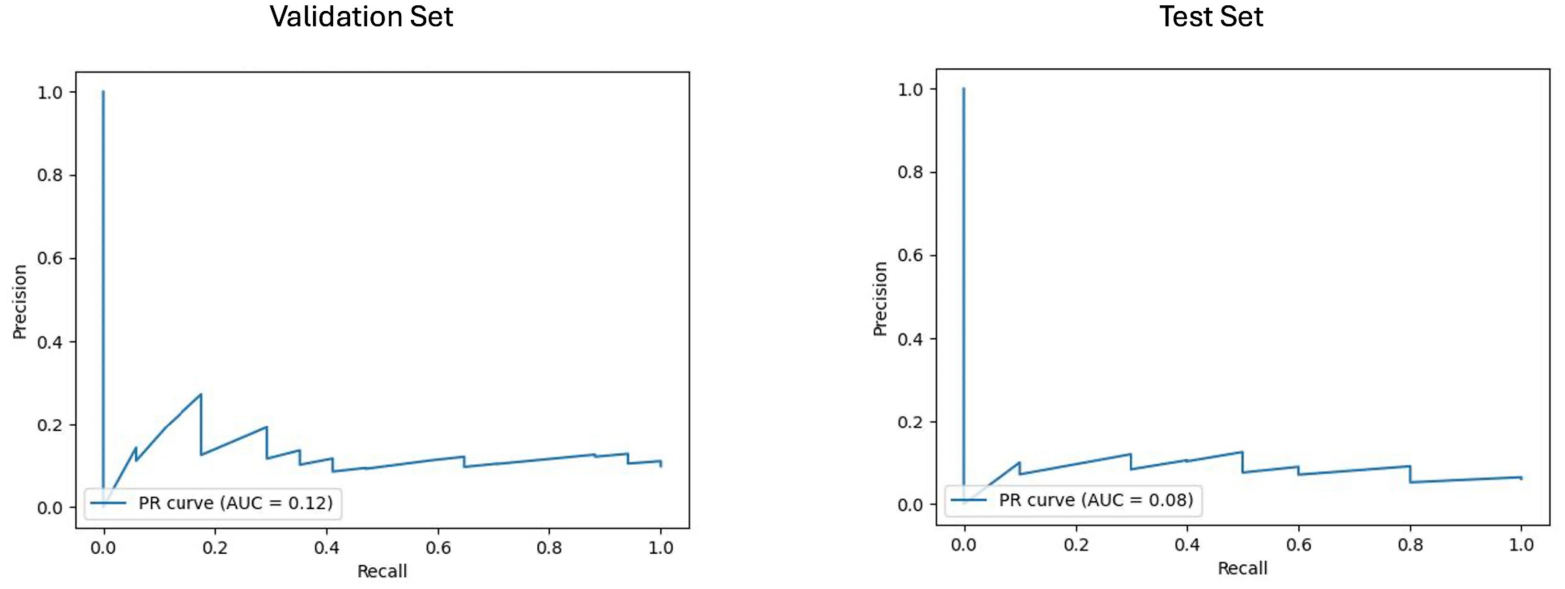
Area under the precision-recall curve (PR AUC) or model performance on clinical validation and test sets. The PR curves illustrate the performance of the best-performing model, the LGBM model trained on clinical features for predicting disability worsening. Validation PR AUC was 0.12, while the test set PR AUC was 0.08.

**Figure 5:**
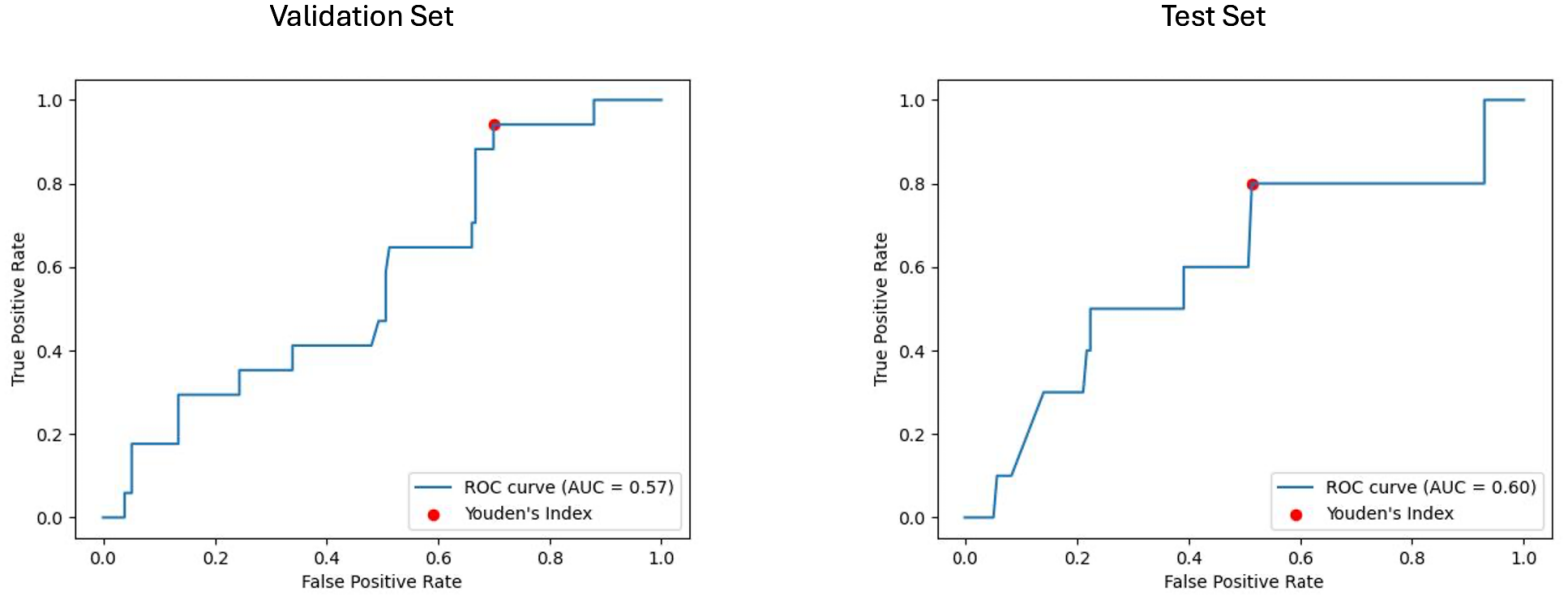
Receiver operating characteristic curve with area under the curve (ROC AUC) for model performance on clinical validation and test set. The ROC curves display the discriminative ability of the best-performing LGBM model trained on clinical features for predicting disability worsening. The ROC AUC for the validation set was 0.57, with a test set ROC AUC of 0.60. The red dots indicate the optimal thresholds determined using Youden’s index, balancing sensitivity and specificity.

The results of the best ML model per prognostic approach are summarized in Table 5. As shown in Figures 4, 5, for the clinical approach, LGBM performed the best by achieving a validation PR AUC of 0.12 and a validation ROC AUC of 0.57. On the test set, it attained a PR AUC of 0.08 and a ROC AUC of 0.6.

**Table 5:** Performance metrics of the best-performing models across approaches, harmonization strategies, and feature subsets.

Approach	Harmonization	Feature subset	Best model	Validation PR AUC	Validation ROC AUC	Test PR AUC	Test ROC AUC
Clinical	Not applicable	Clinical only	LGBM	0.16	0.65	0.08	0.6
Baseline imaging	Harmonized (LongCombat)	Radiomics and clinical features	LGBM	0.25	0.65	0.2	0.64
	Non harmonized	Radiomics and clinical features	BRFC	0.22	0.69	0.13	0.74
Longitudinal imaging	Harmonized (LongCombat)	Radiomics volume features	BRFC	0.41	0.66	0.25	0.48
	Non harmonized	Radiomics features	BRFC	0.32	0.78	0.11	0.69
Combined	Harmonized (LongCombat)	Radiomics features	LOGIT	0.54	0.9	0.06	0.41
	Non harmonized	Radiomics and clinical features	LGBM	0.53	0.91	0.06	0.44

**Figure 6:**
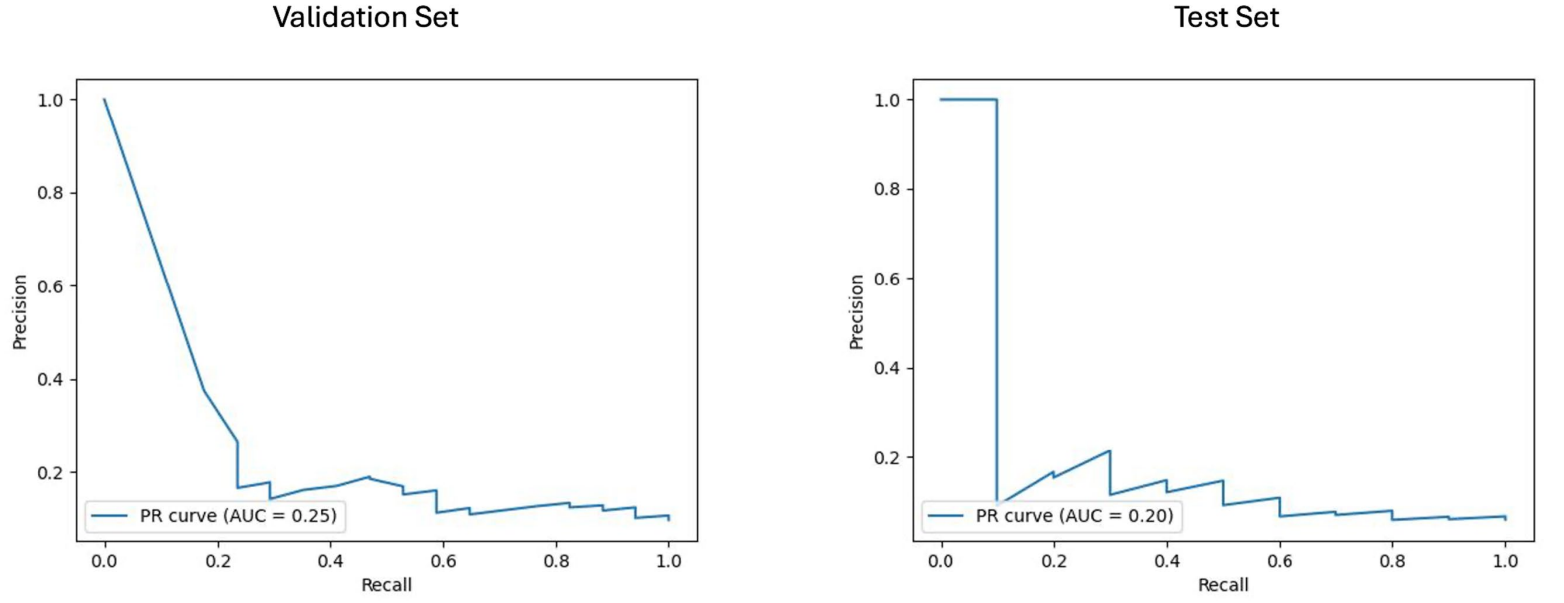
Area under the precision-recall curve (PR AUC) for model performance on baseline imaging validation and test sets with harmonized radiomics and clinical feature subset. The PR curves illustrate the performance of the best-performing model LGBM trained on baseline imaging harmonized radiomics and clinical feature subset for predicting disability worsening. Validation PR AUC was 0.25, while the test set PR AUC was 0.20.

**Figure 7:**
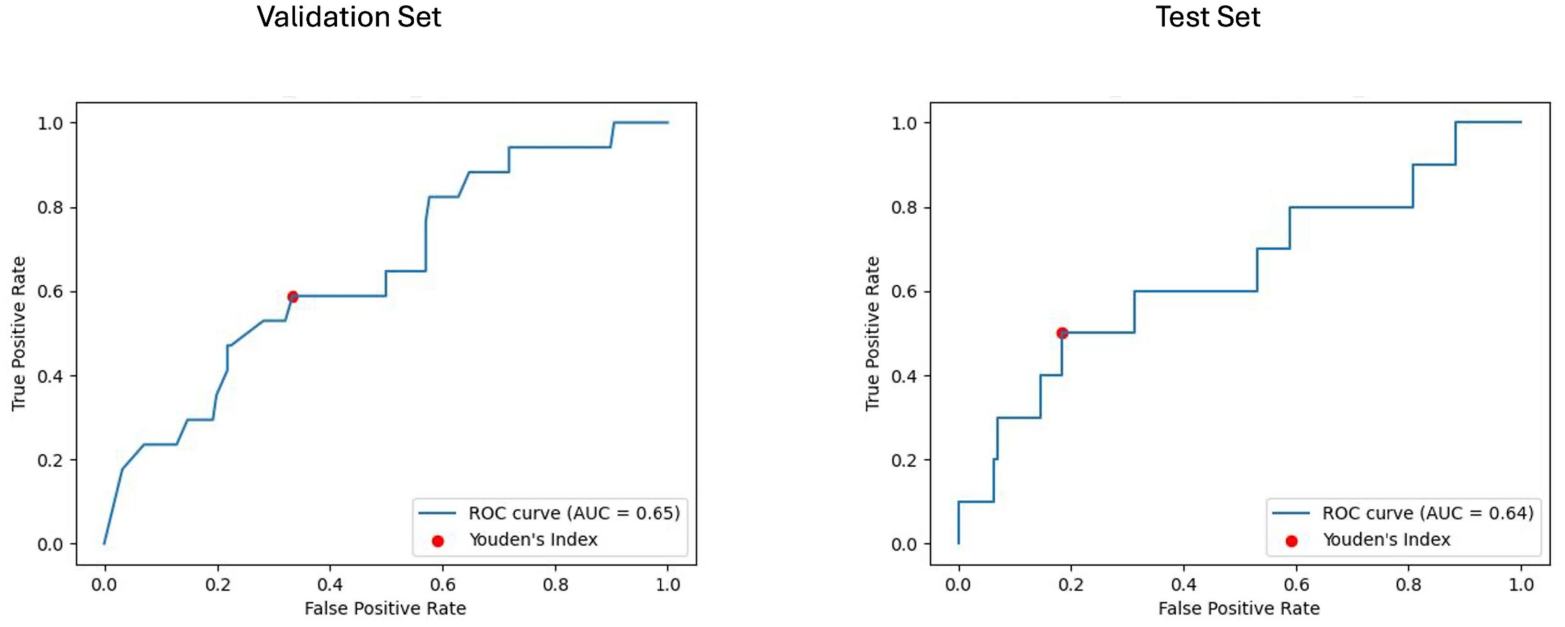
Receiver operating characteristic curve with area under the curve (ROC AUC) for model performance on baseline imaging validation and test sets with harmonized radiomics and clinical feature subset. The ROC curves display the discriminative ability of the best-performing LGBM model trained on baseline imaging, harmonized radiomics, and a clinical feature subset. The ROC AUC for the validation set was 0.65, with a test set ROC AUC of 0.64. The red dots indicate the optimal thresholds determined using Youden’s index, balancing sensitivity and specificity.

For the baseline imaging approach, LGBM performed best in the radiomics and clinical features subset. As shown in Figures 6, 7, LGBM achieved a validation PR AUC of 0.28 and a validation ROC AUC of 0.73. On the test set, it attained a PR AUC of 0.20 and an ROC AUC of 0.64. While the non-harmonized baseline models also generalized well on the test set, they, however, did not achieve better results compared to the baseline harmonized model (see Appendix D).

**Figure 8:**
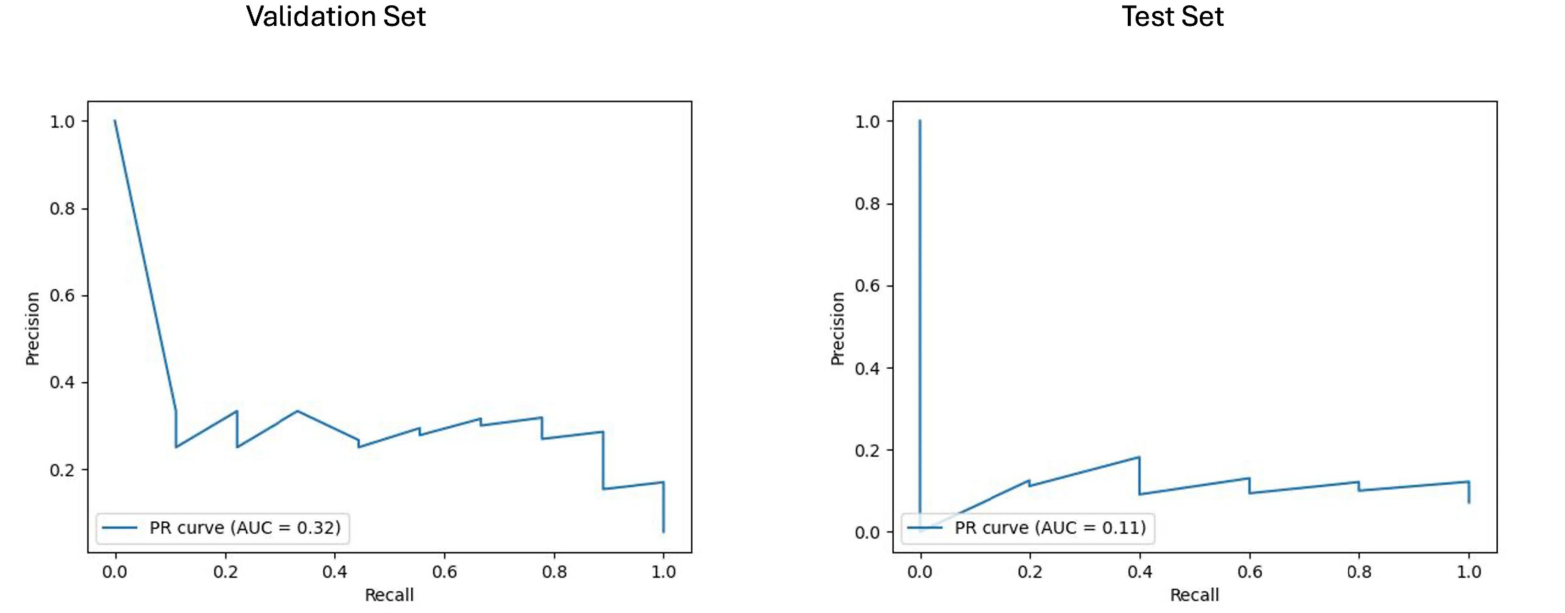
Area under the precision-recall curve (PR AUC) for model performance on longitudinal imaging validation and test sets with a non-harmonized radiomics feature subset. The PR curves depict the performance of the best-performing model, BRFC, for longitudinal imaging non-harmonized radiomics feature subsets. The validation PR AUC is 0.32, and the test PR AUC is 0.11.

**Figure 9:**
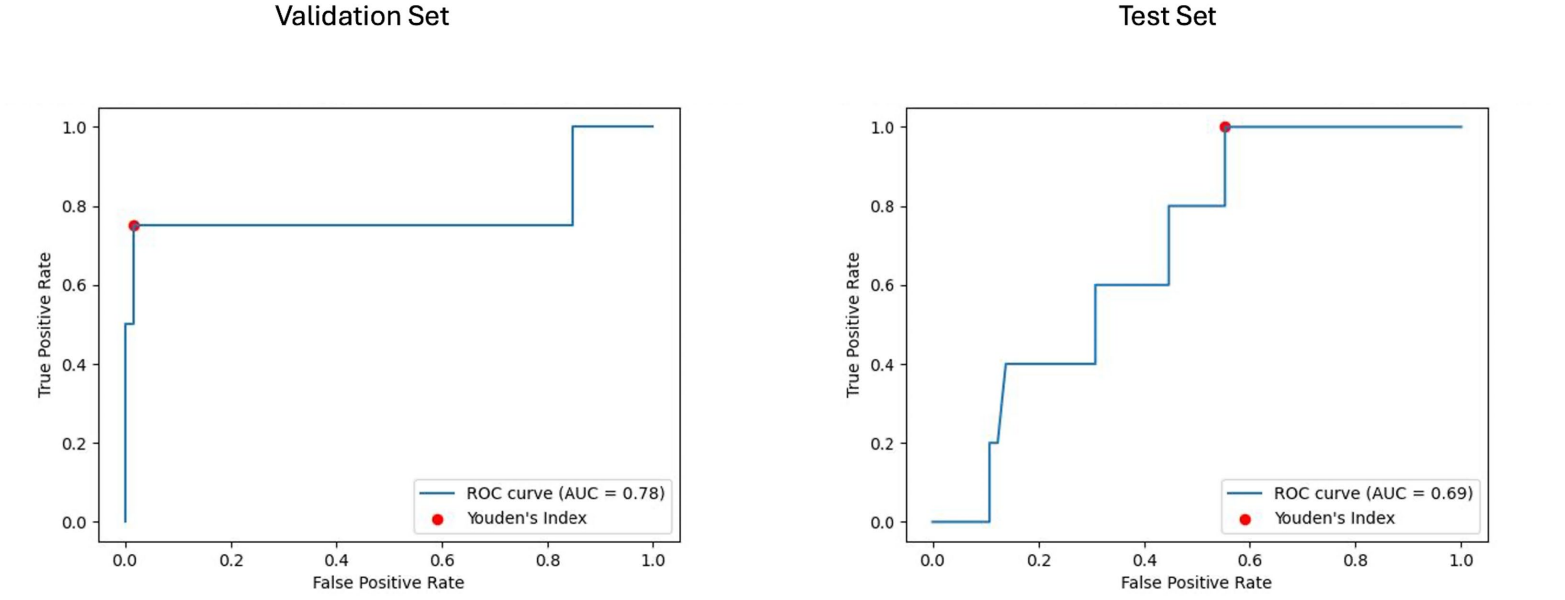
Receiver operating characteristic curve with area under the curve (ROC AUC) for model performance on longitudinal imaging validation and test sets with non-harmonized radiomics feature subset. The ROC curves display the discriminative ability of the best-performing BRFC model for longitudinal imaging non-harmonized radiomics feature subsets. The validation ROC AUC reached 0.78, while the test set ROC AUC was 0.69. The red dots indicate the optimal thresholds determined using Youden’s index, balancing sensitivity and specificity.

For the longitudinal imaging prognostic approach, the BRFC model trained on non-harmonized radiomics features achieved the best results compared to the harmonized approach, with a validation PR AUC of 0.32 and ROC AUC of 0.78, while on the test set, it achieved a PR AUC of 0.11 and an ROC AUC of 0.69 (see Figures 8, 9). The longitudinal imaging harmonized models and the combined models, both harmonized and non-harmonized, did not generalize well on the test set (see Table 5).

Using Youden’s index (J) to determine the optimal threshold, the clinical model achieved a sensitivity of 0.8 and specificity of 0.48 on the test set. For the baseline imaging prognostic approach, the LGBM trained on the harmonized radiomics and clinical features attained a sensitivity of 0.4 and specificity of 0.85 on the test set. Detailed metrics for each approach are shown in Table 6.

**Table 6:** Sensitivity, specificity, precision, and recall metrics for the best-performing models were determined by Youden’s index.

Approach	Harmonization	Feature subset	Best model	Youden’s index	Sensitivity	Specificity	Precision	Recall
Clinical	Not applicable	Clinical only	LGBM	0.03	0.8	0.48	0.09	0.8
Baseline imaging	Harmonized (LongCombat)	Radiomics and clinical features	LGBM	0.0086	0.5	0.81	0.15	0.5
Longitudinal imaging	Non harmonized	Radiomics features	BRFC	0.23	1.0	0.45	0.12	1.0

### SHAP-based feature analysis

**Figure 10:**
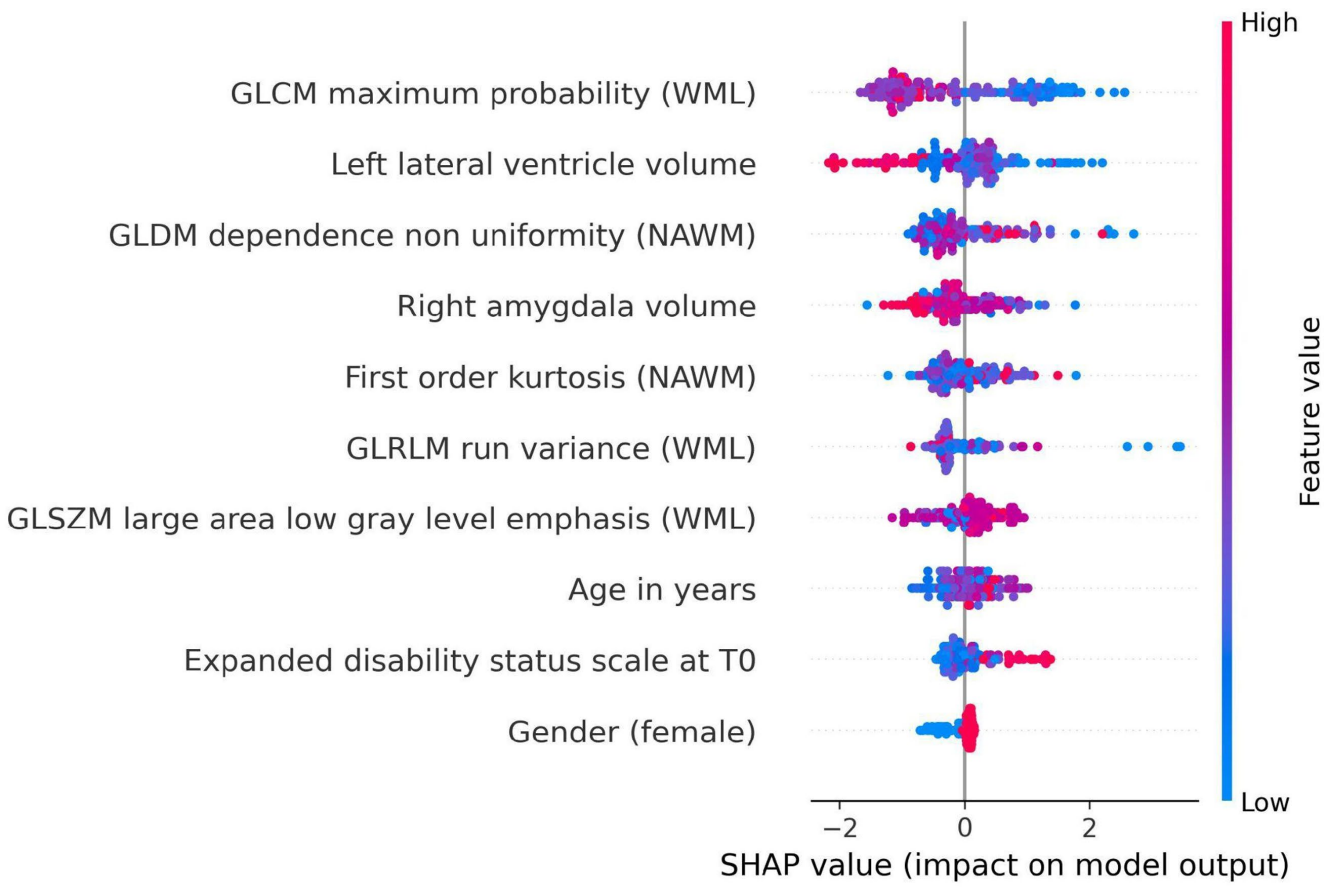
SHAP summary plot for model trained using baseline imaging prognostic approach with harmonized radiomics and clinical feature subset. The SHAP analysis highlights the top contributing features influencing the prediction of disability worsening. Key features include GLCM maximum probability (WML), left lateral ventricle volume, and GLDM dependence non-uniformity (NAWM), reflecting the importance of textural and anatomical characteristics in predicting worsening. Features like gender (female) and age at baseline had relatively lower contributions to the model’s predictions.

The SHAP analysis identified the most influential features contributing to the prediction of disability worsening across the best-performing models in the baseline imaging and longitudinal imaging prognostic approaches. For the baseline imaging prognostic approach with harmonized radiomics and clinical features, as shown in Figure 10, the SHAP analysis revealed GLCM maximum probability (WML), left lateral ventricle volume, and GLDM dependence non-uniformity (NAWM) as the top three features influencing predictions. Features like gender (female) had a lower impact on the model outcome.

**Figure 11:**
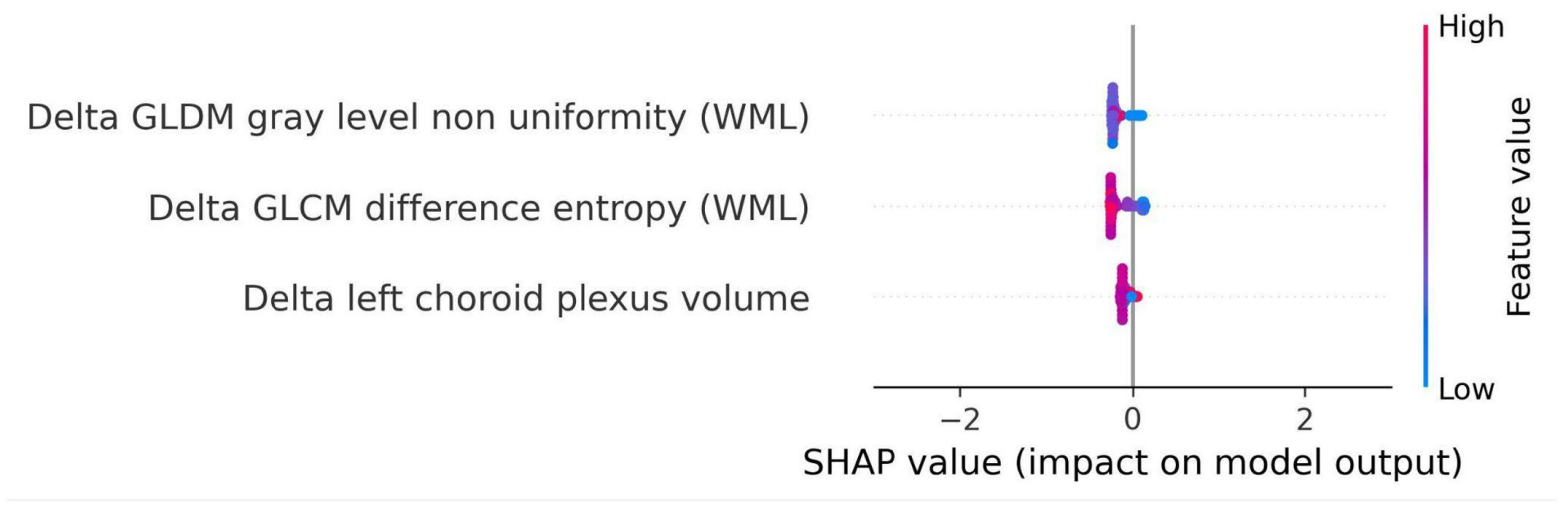
SHAP summary plot for model trained using longitudinal imaging prognostic approach with non-harmonized radiomics feature subset. The SHAP analysis for the delta non-harmonized model identifies delta GLDM gray level non-uniformity (WML) and delta GLCM difference entropy (WML) as the most predictive features for disability worsening. The delta left choroid plexus volume exhibited a lower contribution, underscoring the relative importance of dynamic changes in lesion structure over time.

In the SHAP summary plot (Figures 10, 11), features are ranked by their mean absolute SHAP value, which quantifies their overall importance in the model. The higher the mean absolute SHAP value, the greater the feature’s contribution to predictions across all subjects. The color coding in the plot represents the value of the feature for each subject: red points correspond to higher feature values, while blue points indicate lower feature values. For example, a higher GLCM maximum probability (red points) was associated with a lower likelihood of disability worsening, reflecting its inverse relationship with the outcome.

For the longitudinal imaging approach with a non-harmonized radiomics feature subset, the delta GLDM gray level non-uniformity (WML) and delta GLCM difference entropy (WML) had a higher predictive capability for disability worsening, whereas the delta left choroid plexus volume had a relatively lower contribution to the model’s outcome (Figure 11).

For details on the specific model parameters and Optuna settings, we refer to Appendix I, whereas the details of the CLEAR checklist and RQS are provided in Appendices E, F, respectively.

The results of the permutation testing conducted by shuffling the outcome variable and re-evaluating the models are presented in Appendix J. The findings showed that the performance of the permuted models in all the prognostic approaches was worse compared to the original models.

## Discussion

In this study, we explored the potential of FLAIR MRI-based radiomics and ML techniques on multicentric data to predict disability worsening in people with multiple sclerosis. We deployed three ML models, namely LOGIT, BRFC, and LGBM, across four different prognostic approaches, i.e., clinical, baseline imaging, longitudinal imaging, and combined. Except for the clinical approach, the imaging and combined prognostic approaches further consist of harmonized and non-harmonized feature subsets comprising radiomics volume features, radiomics features without volumes, and radiomics features, as well as radiomics and clinical feature subsets.

Addressing our first research question, whether radiomics-based models can outperform models relying solely on clinical variables, we found that the LGBM model trained on harmonized radiomics and clinical features generalized the best on the test set, achieving a PR AUC of 0.2 and ROC AUC of 0.64 on the test set. As shown in Appendix D, the combination of radiomics and clinical features outperformed both the clinical-only and radiomics-only prognostic approaches, demonstrating the added value of integrating advanced imaging biomarkers with routinely available clinical data. Although a PR AUC of 0.2 may appear low in absolute terms, its interpretation differs from that of the ROC AUC. The baseline for PR AUC is not 0.50 but the prevalence of the positive class. In our cohort, disability worsening occurred in ≈10% of visits, meaning that a random classifier would yield a PR AUC of 0.10 (Maier-Hein et al., 2024). Against this baseline, our model’s PR AUC of 0.20 reflects a doubling of performance relative to chance. This indicates that, across thresholds, the model enriches true disability worsening cases among the high-risk predictions more effectively than random selection would achieve. While modest in absolute terms, such an improvement is meaningful in a severely imbalanced setting, where incremental gains above baseline can translate into clinical utility by prioritizing patients for closer monitoring.

For our second research question, whether radiomics features can predict disability worsening in PwMS, the most generalizable model was the LGBM trained on harmonized data with the radiomics and clinical features subset. As seen in Figure 9, the most influential features constitute textural features from the WML and NAWM. The role of textural features in MS disease worsening has been studied previously (Harrison et al., 2010; Herlidou-Même et al., 2003; Kassner and Thornhill, 2010; Loizou et al., 2010; Loizou et al., 2011; Loizou et al., 2015; Loizou et al., 2020; Meier and Guttmann, 2003; Zhang et al., 2008) and our study further strengthens the notion that textural features can capture the diffuse pathological changes in these areas. The textural features extracted from WML tend to capture the heterogeneity and structural characteristics of the lesions, which can provide a noninvasive means of assessing lesion activity and overall burden, which is critical for MS disease worsening (Zhang et al., 2013). As previously studied, the heterogeneity in voxel intensities corresponds to demyelination, axonal loss, and inflammation in the WML (Zhang et al., 2013; Barkovich, 2000). Furthermore, previous studies have shown that textural heterogeneity can act as relevant biomarkers to predict worsening (Loizou et al., 2011; Tozer et al., 2009). This could be due to the origin of the MRI signal from the endogenous protons, which are affected by the structural changes at the microscopic level in pathology (NAWM and WML), causing magnetic resonance signal variation at the macroscopic scale (Zhang et al., 2013).

The top predictor among the WML textural features in our study was GLCM Maximum Probability (WML), which shows an inverse relationship with disability worsening. This feature essentially measures the most probable co-occurrence of intensity values within an ROI (Haralick et al., 1973). In the case of WML, this would mean that a higher value of GLCM Maximum Probability would indicate a higher degree of homogeneity of intensity values, whereas lower values would indicate lower homogeneity or increased heterogeneity in the WML. Therefore, the more textural heterogeneity a lesion exhibits, the more demyelination and other microstructural changes occur within that lesion (Zhang et al., 2013; Barkovich, 2000). The textural features extracted from the NAWM were also deemed as predictive features. They represent possible diffuse pathological changes such as gliosis or early demyelination, which are not visible to the naked eye on MRI. This is in line with the literature (Zhang, 2012; Zhang et al., 2009).

In addition to textural features, anatomical volumes such as left lateral ventricle volume and right amygdala volume were also deemed useful in our study. Although ventricular enlargement corresponds to brain atrophy, which corresponds further to disability worsening, in our study, the ventricular enlargement exhibited a negative correlation with disability worsening, unlike previous studies (Genovese et al., 2019; Jakimovski et al., 2020; Zivadinov et al., 2019). As shown in Appendix M, this negative correlation can be attributed to cases with advanced atrophy (high left lateral ventricle volume) being labeled as non-progressive, as their baseline disability score (EDSS_T0) is already high, leaving little room for measurable worsening within the two-year follow-up period.

Conversely, a larger right amygdala volume was associated negatively with disability worsening, pointing to the possible role of the limbic system in preserving cognitive and neurological function in MS. Lastly, the clinical features did not have as much of a higher influence as the others, but they remained important predictors nevertheless. The highest being the age at baseline exhibiting a positive correlation with disability worsening (Kalnina et al., 2024).

For our last research question, i.e., whether short-term changes in MRI features can predict disability worsening, we used the longitudinal imaging prognostic approach in an attempt to capture temporal changes and exploit its use to make our models robust. We found that the BRFC trained on the non-harmonized radiomics feature subset achieved a PR AUC of 0.11 and ROC AUC of 0.69 on the test set. However, looking at the features and their corresponding SHAP values, we observed that the selected features, corresponding to the dynamic changes in lesion structure over time, exhibited a lower predictive power compared to the harmonized features selected in the baseline imaging approach. This could be due to the short temporal window between the baseline and follow-up and a reduction in the dataset, which inhibits their capability to fully capture short-term changes to explain disability worsening in PwMS.

To further validate our findings and eliminate the risk of overfitting, the permutation results, presented in Appendix J, indicate that the permuted models’ performance was notably worse than the original models. This indicates that the predictive power of our models is driven by meaningful patterns in the data rather than random noise or spurious correlations. Furthermore, the poor performance of the permuted models validates the robustness of our approach, as any enhancement in prediction performance observed in the original models cannot be attributed to chance.

Interestingly, the combined prognostic approach did not yield a generalizable predictive performance, suggesting that the baseline imaging prognostic approach is sufficient to capture the majority of relevant information for predicting disability worsening. The integration of longitudinal imaging features may have introduced noise, diluting the predictive signal of the more robust baseline features. These results underscore the need for careful feature selection and refined temporal analysis to optimize combined approaches.

This study brings important advancements compared to the existing literature. By leveraging multicentric data from two centers with diverse MRI acquisition protocols, it enhances the generalizability of findings. The robust preprocessing pipeline, including super-resolution reconstruction and longitudinal ComBat harmonization, attempted to ensure consistency in imaging data across sites and protocols. Additionally, the use of SHAP analysis provided interpretable insights into feature importance, offering a deeper understanding of the role of radiomics in predicting MS worsening. While MRI, unlike computed tomography, is inherently non-quantitative, our study, similar to previous work (Lavrova et al., 2021), demonstrates the potential of radiomics features in capturing subtle pathological changes. The selection of radiomics features from the WML and NAWM, coupled with radiomics and clinical features, further enhances the promise radiomics holds to bridge the gap between radiological findings and clinical outcomes, also known as the clinic-radiological paradox (Uitdehaag, 2018).

However, certain limitations must be acknowledged. The small number of worsening disability worsening cases translated into a high-class imbalance, which posed challenges despite the use of weighted adjustments. The reliance on reconstructed images without ground truth and the absence of T1-weighted sequences may have affected segmentation and feature quality. While initially, we performed ML analysis where DS2 was kept as a completely held-out external set, the models tended to generalize poorly on it (see Appendix G). Although longitudinal ComBat harmonization attempts to mitigate scanner and site variability, its ability to preserve subtle predictive patterns and address batch effects warrants further validation. Finally, the retrospective design may introduce selection bias and asymmetry, limiting the generalizability of these findings to broader populations.

Future studies should address these limitations by incorporating larger, multicentric, balanced datasets with higher-resolution MRI and ground-truth labels. Expanding the temporal window for delta radiomics and integrating advanced imaging modalities, such as diffusion-weighted imaging, may enhance the predictive power of radiomics. Moreover, exploring the role of other clinical variables, such as disease-modifying therapy, disease duration, and lesion topography (for example through periventricular, juxtacortical, and infratentorial labels or atlas-based lesion load), alongside imaging biomarkers could provide a more comprehensive understanding of worsening mechanisms in MS. Finally, deep radiomics with pre-trained foundation models can be deployed to see whether a deep learning algorithm might be able to uncover patterns that the traditional ML algorithm with hand-crafted radiomics might have failed to capture.

## Conclusion

This study highlights the potential of FLAIR MRI-based radiomics combined with ML to predict two-year disability worsening in PwMS. We demonstrated that models combining radiomics and clinical features outperform clinical-only models. Furthermore, we found that radiomics features from WML and NAWM and routine clinical features from the baseline imaging prognostic approach emerged as predictors, reinforcing their diagnostic value. However, the longitudinal imaging approach demonstrated limited predictive power, emphasizing the need for refined temporal analysis. Future work should address class imbalance, enhance feature quality, and explore advanced imaging modalities to further advance MS worsening prediction.

## Data Availability

The datasets presented in this study are not publicly available due to institutional and privacy restrictions. Reasonable requests to access the data can be directed to the corresponding author and will be considered in line with institutional and ethical guidelines. The authors do not rule out the possibility of making the dataset publicly available in the future.

## Footnotes

^1^ www.statistical-modelling.de/lst.html

^2^ http://www.fil.ion.ucl.ac.uk/spm
