# Supplementary material for: Leveraging hand-crafted radiomics on multicenter FLAIR MRI for predicting disability worsening in people with multiple sclerosis
Source: Front Neurosci. 2025 Oct 29;19:1610401. doi: 10.3389/fnins.2025.1610401 (PMC12607017; doi:10.3389/fnins.2025.1610401)
Supplement: Supplementary file 1 [file Data_Sheet_1.PDF]

Supplementary Information

Appendix A: T2-W FLAIR MRI acquisition details

For DS1-Pelt:

N sessions	Scanner	Protocol	Acquisition type	Images per session	Repetition time	Echo time	Inversion time	Slice thickness	Slice spacing	Pixel spacing
487	Philips Achieva 1.5T	A	2D	2	6000 ms	120 ms	2000 ms	5 mm	6 mm	0.72 - 0.90 mm
64	Philips Achieva 1.5T	B	3D	3	4800 ms	321 - 363 ms	1660 ms	3 mm	3 mm	0.98 mm
79	Philips Achieva 1.5T	C/CsT1	3D	1	4800 ms	323 - 356 ms	1660 ms	1.2 mm	0.6 mm	0.98 mm

For DS2-Zuyderland:

N sessions	Scanner	Protocol	Acquisition type	Images per session	Repetition time	Echo time	Inversion time	Slice thickness	Slice spacing	Pixel spacing
9	Philips Achieva 1.5T	D	2D	1	11000 ms	140 ms	2800 ms	5 mm	6 mm	0.9 mm
73	Philips Achieva 1.5T	A	2D	2	9000 - 11000 ms	140 ms	2800 ms	5 mm	6 mm	0.8 - 0.9 mm
2	Philips Achieva 1.5T	C	3D	1	4800 ms	319 ms	1660 ms	1 mm	1 mm	0.96 mm

2	Philips Achieva 3T	D	2D	1	11000 ms	125 ms	2800 ms	5 mm	5.5 mm	0.45 mm
1	Philips Ingenia 3T	C	3D	1	4800 ms	300 ms	1650 ms	0.9 mm	0.9 mm	0.24 mm
2	SIEMENS Aera 1.5T	A	2D	2	9000 ms	86 - 90 ms	2500 ms	3 - 5mm	3.9 - 6.5 mm	0.72 - 0.73 mm
15	SIEMENS Avanto 1.5T	D	2D	1	8000 - 11900 ms	102 - 125 ms	2500 ms	3 - 5 mm	3 - 7 mm	0.45 - 1 mm
21	SIEMENS Avanto 1.5T	A	2D	2	6000 - 11900 ms	102 - 125 ms	2500 ms	3 - 5 mm	3 - 7 mm	0.45 - 1 mm
1	SIEMENS Avanto_fit 1.5T	D	2D	1	9000 ms	85 ms	2500 ms	4 mm	4.4 mm	0.72 mm
35	SIEMENS Avanto_fit 1.5T	A	2D	2	6000 - 9000 ms	85 - 107 ms	2020 - 2500 ms	4 - 5 mm	4.4 - 5.5 mm	0.45 - 0.9 mm
1	SIEMENS Avanto_fit 1.5T	CsT1	3D	1	5000 ms	381 ms	1800 ms	1 mm	1 mm	1 mm
1	SIEMENS MAGNETOM EXPERT plus 0.95T	A	2D	2	9000 ms	105 ms	2223 ms	5 mm	6.5 mm	0.98 mm
2	SIEMENS Skyra 3T	D	2D	1	9400 ms	78 - 112 ms	2500 ms	3 mm	3 mm	1 mm
10	SIEMENS Skyra 3T	A	2D	2	9000 ms	81 ms	2500 ms	4 mm	4.4 - 5.2 mm	0.72 mm
7	SIEMENS Skyra 3T	CsT1	3D	1	5000 ms	384 - 388 ms	1800 ms	0.9 - 1 mm	0.9 - 1 mm	0.9 - 1 mm
2	SIEMENS Symphony	A	2D	2	9000 ms	106 - 128 ms	2500 ms	3 - 5 mm	3.9 - 6.5 mm	0.45 - 0.9 mm

	1.5T									

Appendix B: Anatomical brain structures segmented by SAMSEG

Number	Feature
1	left cerebral cortex volume
2	right cerebral cortex volume
3	left cerebral white matter volume
4	right cerebral white matter volume
5	left cerebellum cortex volume
6	right cerebellum cortex volume
7	left cerebellum white matter volume
8	right cerebellum white matter volume
9	right amygdala volume
10	left amygdala volume
11	right hippocampus volume
12	left hippocampus volume
13	left accumbens area volume
14	right accumbens area
15	left putamen volume
16	right putamen volume
17	right pallidum volume
18	left left pallidum volume
19	left caudate volume
20	right caudate volume
21	right thalamus volume

22	left thalamus volume
23	right choroid plexus volume
24	left choroid plexus volume
25	right ventral diencephalon volume
26	left ventral diencephalon volume
27	right inferior lateral ventricle volume
28	left inferior lateral ventricle volume
29	fourth ventricle volume
30	third ventricle volume
31	left lateral ventricle volume
32	right lateral ventricle volume
33	cerebrospinal fluid volume
34	brain stem volume
35	cerebral grey matter cortex volume
36	cerebral white matter volume
37	ventricle volume
38	total white matter lesion volume
39	intra cranial volume
40	fifth ventricle volume
41	unknowns volume

Appendix C: Features selected for all prognostic approaches and feature subsets

Approach	Harmonisation	Feature Subset	Total Features	Non inter-correlated Features	N	Selected Features
Clinical	Not applicable	Clinical only	3	-	3	EDSS_T0, clinical age in years, gender (female)
Baseline imaging	Harmonised	Radiomics volume features	41	41	4	right hippocampus volume, left lateral ventricle volume, right thalamus volume, right amygdala volume
		Radiomics features without volumes	200	103	6	GLRLM run variance (WML), firstorder kurtosis (NAWM), GLCM maximum probability (WML), GLSZM large area low gray level emphasis (WML), shape minor axis length (NAWM), GLDM dependence non uniformity (NAWM)
		Radiomics	10 (selected features from radiomics volume features and radiomics features without volumes)	10 (selected features from radiomics volume features and radiomics features without volumes)	7	GLRLM run variance (WML), GLCM maximum probability (WML), firstorder kurtosis (NAWM), left lateral ventricle volume, GLDM dependence non uniformity (NAWM), right amygdala volume, GLSZM large area low gray level emphasis (WML)
		Radiomics and clinical features	13 (selected features from radiomics	13 (selected features from radiomics along with clinical features)	10	GLRLM run variance (WML), GLCM maximum probability (WML), firstorder kurtosis (NAWM), left lateral ventricle volume, GLDM dependence

			along with clinical features)			non uniformity (NAWM), right amygdala volume, GLSZM large area low gray level emphasis (WML), EDSS_T0, clinical age in years, gender (female)
	Non harmonised	Radiomics volume features	41	41	12	left lateral ventricle volume, right accumbens area volume, left thalamus volume, right thalamus volume, left left pallidum volume, right amygdala volume, left choroid plexus volume, left caudate volume, left ventral diancephalon volume, left accumbens area volume, left hippocampus volume, right inferior lateral ventricle volume
		Radiomics features without volumes	200	98	19	GLRLM run variance (WML), firstorder minimum (WML), first order 10 percentile (NAWM), GLCM cluster shade (WML), shape minor axis length (NAWM), GLDM large dependence high gray level emphasis (WML), GLDM dependence entropy (NAWM), shape maximum 2D diameter slice (WML), shape maximum 2D diameter column (NAWM), shape major axis length (WML), shape sphericity (NAWM), GLRLM run entropy (NAWM), GLCM joint energy (WML), shape surface volume ratio (NAWM), GLCM maximum probability (WML), shape flatness (NAWM), firstorder mean (WML),

						GLSZM size zone non uniformity (WML), GLCM joint entropy (WML)
		Radiomics	31 (selected features from radiomics volume features and radiomics features without volumes)	31 (selected features from radiomics volume features and radiomics features without volumes)	10	GLRLM run variance (WML), left thalamus volume, left lateral ventricle volume, firstorder minimum (WML), right accumbens area volume, right thalamus volume, left left pallidum volume, right amygdala volume, GLSZM size zone non uniformity (WML), shape minor axis length (NAWM)
		Radiomics and clinical features	34 (selected features from radiomics along with clinical features)	34 (selected features from radiomics along with clinical features)	13	GLRLM run variance (WML), left thalamus volume, left lateral ventricle volume, firstorder minimum (WML), right amygdala volume, right accumbens area volume, right thalamus volume, left left pallidum volume, GLSZM size zone non uniformity (WML), shape minor axis length (NAWM), EDSS_T0, clinical age in years, gender (female)
Longitudinal imaging	Harmonised	Radiomics volume features	41	38	1	delta right cerebellum cortex volume
		Radiomics features without volumes	200	179	1	delta GLCM difference entropy (WML)
		Radiomics	2 (selected features	2 (selected features from	2	delta Right cerebellum cortex volume, delta GLCM difference

			from radiomics volume features and radiomics features without volumes)	radiomics volume features and radiomics features without volumes)		entropy (WML)
		Radiomics and clinical features	5 (selected features from radiomics along with clinical features)	5 (selected features from radiomics along with clinical features)	5	delta right cerebellum cortex volume, delta GLCM difference entropy (WML), EDSS_T0, delta clinical age in years, gender (female)
	Non harmonised	Radiomics volume features	41	38	1	delta left choroid plexus volume
		Radiomics features without volumes	200	178	2	delta GLCM difference entropy (WML), delta GLDM gray level non uniformity (WML)
		Radiomics	3 (selected features from radiomics volume features and radiomics features without volumes)	3 (selected features from radiomics volume features and radiomics features without volumes)	3	delta GLCM difference entropy (WML), delta GLDM gray level non uniformity (WML), delta left choroid plexus volume
		Radiomics and clinical	6 (selected features	6 (selected features from	6	delta GLCM difference entropy (WML), delta GLDM gray level

		features	from radiomics along with clinical features)	radiomics along with clinical features)		non uniformity (WML), delta left choroid plexus volume, EDSS_T0, delta clinical age in years, gender (female)
Combined	Harmonised	Radiomics volume features	82	77	5	left thalamus volume, delta Left cerebellum cortex volume, delta right hippocampus volume, delta right thalamus volume, right thalamus volume
		Radiomics features without volumes	400	222	1	delta GLSZM_large area high gray level emphasis (NAWM)
		Radiomics	6 (selected features from radiomics volume features and radiomics features without volumes)	6 (selected features from radiomics volume features and radiomics features without volumes)	6	left thalamus volume, delta Left cerebellum cortex volume, delta right hippocampus volume, delta right thalamus volume, right thalamus volume, delta GLSZM_large area high gray level emphasis (NAWM)
		Radiomics and clinical features	8 (selected features from radiomics along with clinical features)	8 (selected features from radiomics along with clinical features)	8	left thalamus volume, delta Left cerebellum cortex volume, delta right hippocampus volume, delta right thalamus volume, right thalamus volume, delta GLSZM_large area high gray level emphasis (NAWM), clinical_age, delta clinical_age, gender (female)
	Non harmonised	Radiomics volume	82	77	6	delta right caudate volume, right thalamus volume, delta

		features				left cerebellum cortex volume, left thalamus volume, delta right thalamus volume, brain stem volume
		Radiomics features without volumes	400	216	2	delta GLDM large dependence high gray level emphasis (NAWM),<br>delta GLSZM gray level non uniformity (NAWM)
		Radiomics	8 (selected features from radiomics volume features and radiomics features without volumes)	8 (selected features from radiomics volume features and radiomics features without volumes)	7	delta GLSZM gray level non uniformity (NAWM), left thalamus volume, delta GLDM large dependence high gray level emphasis (NAWM), right thalamus volume, brain stem volume, delta right thalamus volume, delta right caudate volume
		Radiomics and clinical features	11 (selected features from radiomics along with clinical features)	11 (selected features from radiomics along with clinical features)	11	delta GLSZM gray level non uniformity (NAWM), left thalamus volume, delta GLDM large dependence high gray level emphasis (NAWM), right thalamus volume, brain stem volume, delta right thalamus volume, delta right caudate volume, EDSS_T0, clinical age in years, delta clinical age in years, gender (female)

Appendix D : Performances of best models trained per feature subset

Approach	Harmonisation	Feature Subset	Best Model	Validation PR	Validation AUC	Test PR	Test AUC
Clinical	Not applicable	Clinical only	LGBM	0.16	0.65	0.08	0.6
Baseline imaging	Harmonised (LongCombat)	Radiomics volume features	LOGIT	0.13	0.68	0.08	0.56
		Radiomics without volumes	LOGIT	0.2	0.64	0.12	0.74
		Radiomics	BRFC	0.14	0.76	0.13	0.67
		Radiomics and clinical	LGBM	0.25	0.65	0.2	0.64
	Non harmonised	Radiomics volume features	LGBM	0.13	0.59	0.09	0.62
		Radiomics without volumes	BRFC	0.11	0.61	0.16	0.74
		Radiomics	BRFC	0.18	0.72	0.2	0.63
		Radiomics and clinical	BRFC	0.22	0.69	0.13	0.74
Longitudinal imaging	Harmonised (LongCombat)	Radiomics volume features	BRFC	0.41	0.66	0.25	0.48

		Radiomics without volumes	LOGIT	0.30	0.52	0.06	0.49
		Radiomics	LOGIT	0.30	0.51	0.05	0.38
		Radiomics and clinical	LOGIT	0.30	0.51	0.06	0.41
	Non harmonised	Radiomics volume features	BRFC	0.08	0.73	0.27	0.67
		Radiomics without volumes	BRFC	0.32	0.8	0.14	0.66
		Radiomics	BRFC	0.32	0.78	0.11	0.69
		Radiomics and clinical	LOGIT	0.31	0.64	0.08	0.53
Combined	Harmonised (LongCombat)	Radiomics volume features	LGBM	0.36	0.81	0.06	0.45
		Radiomics without volumes	LGBM	0.21	0.44	0.07	0.52
		Radiomics	LOGIT	0.54	0.9	0.06	0.41
		Radiomics and clinical	BRFC	0.46	0.68	0.06	0.45
	Non harmonised	Radiomics volume features	LGBM	0.33	0.72	0.05	0.38

		Radiomics without volumes	BRFC	0.20	0.42	0.05	0.41
		Radiomics	LGBM	0.31	0.65	0.05	0.30
		Radiomics and clinical	LGBM	0.53	0.91	0.06	0.44

Appendix E Radiomics Check-list (CLEAR) - attached seperately

Check_list_Radiomics_only.docx

Appendix F Radiomics Quality Score (attached separately)

RQS - Radiomics.world.pdf

Appendix G Model Results with keeping ZMC as external

**Approach**	**Harmonisation**	**Feature Subset**	**Best Model**	**Validation PR AUC**	**Validation ROC AUC**	**Test PR AUC**	**Test ROC AUC**
Clinical	Not applicable	Clinical only	LOGIT	0.1	0.65	0.09	0.32
Baseline imaging	Harmonised (LongCombat)	Radiomics	LOGIT	0.1	0.74	0.13	0.47
	Non harmonised	Radiomics	LGBM	0.16	0.6	0.13	0.44
Longitudinal imaging	Harmonised (LongCombat)	Radiomics	LOGIT	0.15	0.73	0.11	0.48
	Non harmonised	Radiomics features without volume	BRFC	0.17	0.63	0.08	0.45
Combined	Harmonised (LongCombat)	Radiomics and clinical	LOGIT	0.18	0.59	0.09	0.46
	Non harmonised	Radiomics and clinical	BRFC	0.17	0.53	0.08	0.45

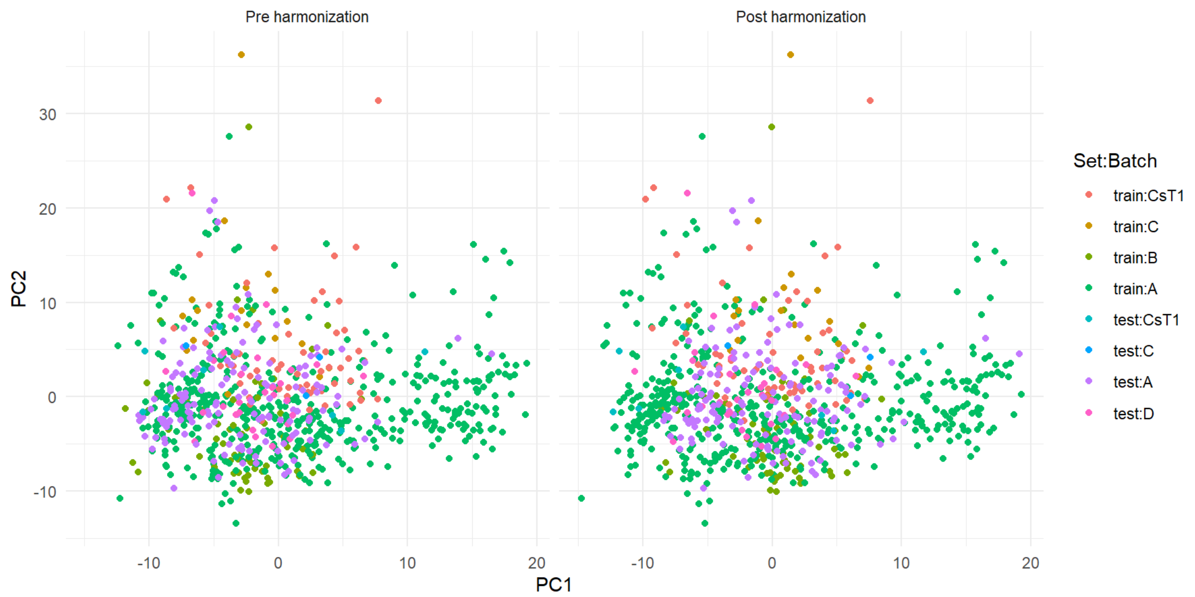

Appendix H: Principal Component Analysis (PCA) Before and After Longitudinal ComBat Harmonisation.
The PCA plots illustrate the distribution of radiomic features across different batches and datasets (train and test) before (left) and after (right) harmonisation using longitudinal ComBat.

Appendix I: Best Models Parameters

Approach	Harmonisation	Feature Subset	Best Model	Model Parameters
Clinical	Not applicable	Clinical only	LGBM	weight_class_1: 2.861272625103398<br>n_estimators: 149<br>max_depth: 7<br>learning_rate: 0.04500730948996164<br>num_leaves: 26
Baseline imaging	Harmonised (Long Combat)	Radiomics and clinical	LGBM	weight_class_1: 2.724695499698599<br>n_estimators: 158<br>max_depth: 10<br>learning_rate: 0.05558146280454876<br>num_leaves: 20
Longitudinal imaging	Non Harmonised	Radiomics	BRFC	weight_class_1: 2.3572082945950172<br>n_estimators: 36<br>max_depth: 4<br>min_samples_split: 4<br>min_samples_leaf: 2

Appendix J: Permutation results achieved on the best approach and feature subset combination

**Approach**	**Harmonisation**	**Feature Subset**	**Best Model**	**Validation PR AUC**	**Validation ROC AUC**
Clinical	Not applicable	Clinical only	LGBM	0.08 ± 0.03	0.45 ± 0.06
Baseline imaging	Harmonised (Long Combat)	Radiomics and clinical	LGBM	0.007 ± 0.01	0.48 ± 0.05
Longitudinal imaging	Non Harmonised	Radiomics	BRFC	0.03 ± 0.04	0.46 ± 0.1

Appnedix K: Number of Radiomics Features Per Class

**Feature Class**	**Number of Features Extracted per ROI**
shape	14
first-order statistics (FO)	18
gray-level co-occurrence matrix (GLCM)	22
gray-level run length matrix (GLRLM)	16
gray-level size zone matrix (GLSZM)	16
gray-level dependence matrix (GLDM)	14

Appendix L: Distribution plots of disability progression in selected features of both Baseline and Longitudinal prognostic approach

Baseline imaging features plots.

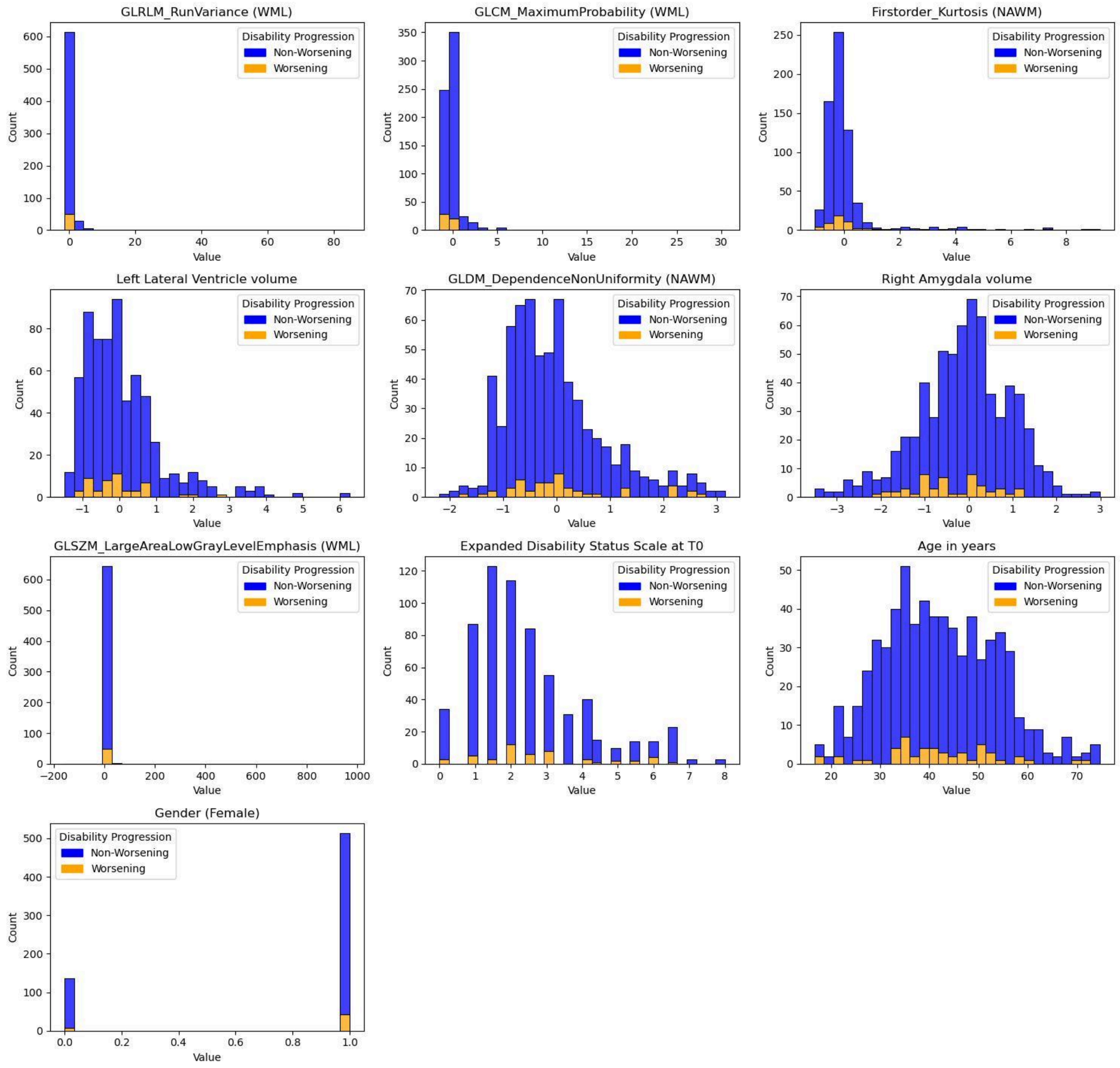

Longitudinal imaging Features Plots:

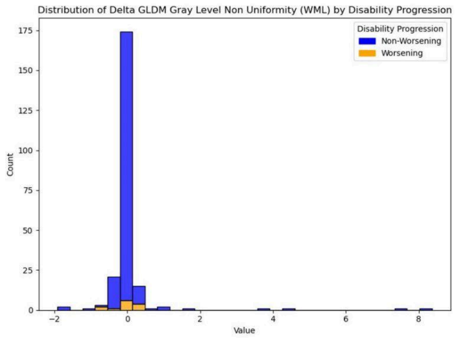
Distribution of Delta GLDM Gray Level Non Uniformity (WML) by Disability Progression
Disability Progression
Non-Worsening
Worsening
Count
Value

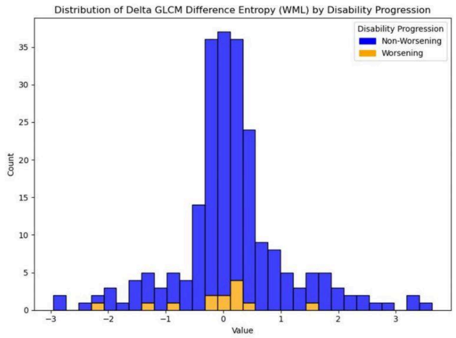
Distribution of Delta GLCM Difference Entropy (WML) by Disability Progression
Disability Progression
Non-Worsening
Worsening
Count
Value

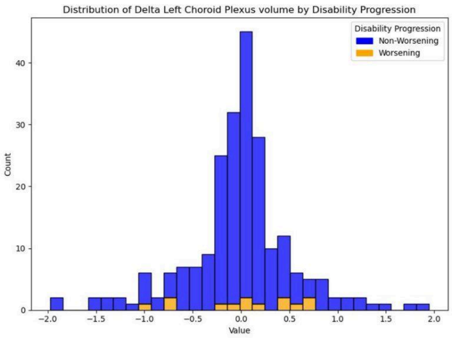
Distribution of Delta Left Choroid Plexus volume by Disability Progression
Disability Progression
Non-Worsening
Worsening
Count
Value

Appendix M: Left Lateral Ventricle Volume vs. Baseline Disability Score (EDSS_T0) by Disability Progression Status.

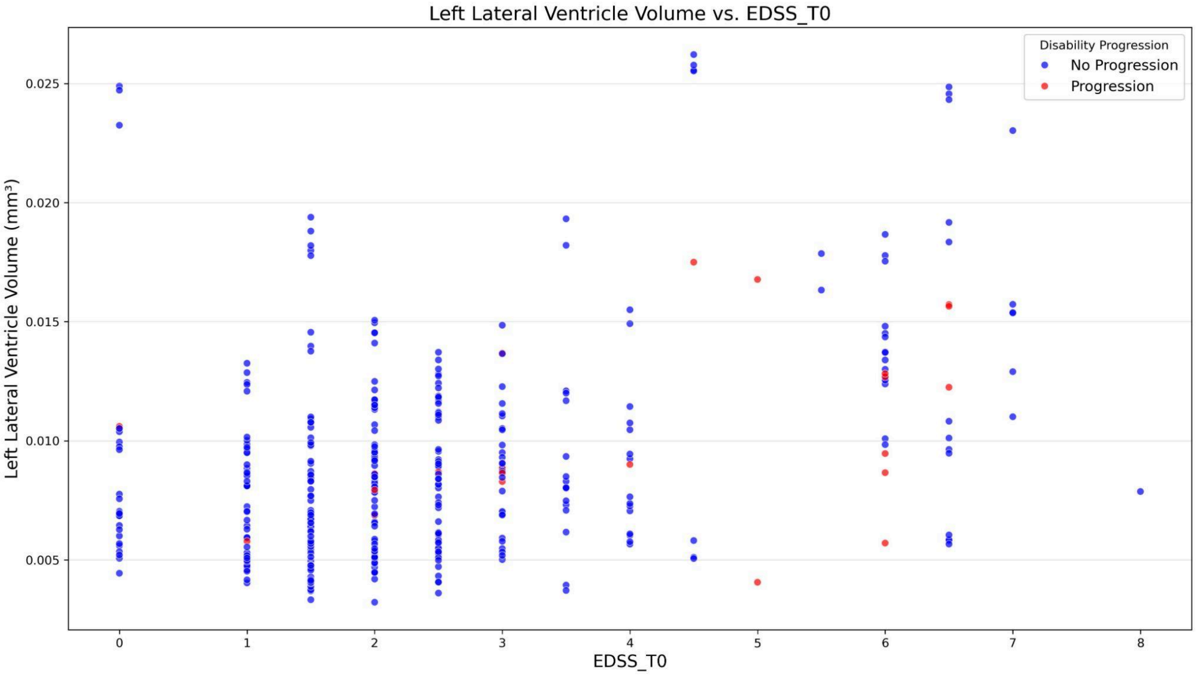

Each point represents an individual case, colour-coded by progression status (blue for no progression, red for progression).
